# Meteorin-like regulates cardiac repair via macrophage modulation following myocardial infarction

**DOI:** 10.1007/s00395-026-01203-7

**Published:** 2026-07-31

**Authors:** G. Ferrer-Curriu, A. Blasco-Roset, A. Navarro-Gascon, C. Soler-Botija, F. J. Godoy-Nieto, M. Monguió-Tortajada, R. Cereijo, T. Quesada-Lopez, F. Rueda, S. Roura, C. Gálvez-Montón, F. Villarroya, A. Bayés-Genís, A. Planavila

**Affiliations:** 1 ICREC Research Program, Germans Trias i Pujol Research Institute (IGTP), Barcelona, Spain; 2 Heart Institute (iCOR) Germans Trias i Pujol University Hospital, Badalona, Barcelona, Spain; 3 CIBER Cardiovascular (CIBERCV), Instituto de Salud Carlos III, Madrid, Spain; 4 Departament de Bioquímica i Biomedicina Molecular, Institut de Biomedicina (IBUB), Universitat de Barcelona and CIBER Fisiopatología de la Obesidad y Nutrición, Avda Diagonal 643, 08028 Barcelona, Spain; 5 Department of Immunobiology, University of Lausanne, 1066 Epalinges, Switzerland; 6 Faculty of Medicine, University of Vic-Central University of Catalonia (UVic-UCC), Vic/Manresa, Barcelona, Spain; 7 Department of Medicine, Autonomous University of Barcelona, Barcelona, Spain

**Keywords:** Meteorin-like, Macrophages, Myocardial infarction, Cardiac repair, Oncostatin M

## Abstract

After myocardial infarction (MI), the heart undergoes a reparative process that includes an initial acute inflammatory phase followed by a subsequent reparative phase. The transition between these phases is crucial for cardiac recovery, but the key factors remain unclear. Meteorin-like (Metrnl) promotes anti-inflammatory/reparative macrophage polarization in the myocardium, yet its role in the acute phase post-MI is unknown. We observed that macrophages infiltrating the ischemic myocardium produced elevated levels of Metrnl in both the heart and circulation 4 days post-MI. The absence of Metrnl in *Metrnl⁻/⁻* mice altered myocardial healing and remodeling, with an increased presence of macrophages with a more pro-inflammatory phenotype. Conversely, cardiac Metrnl overexpression restored myocardial repair and promoted a shift toward a more anti-inflammatory/reparative macrophage phenotype. Mechanistically, Metrnl regulated macrophage-dependent production of Oncostatin M (Osm), a key cytokine in the early inflammatory phase post-MI that induces cardiomyocyte production of Reg3β. Reg3β, in turn, limits pro-inflammatory macrophage activation and polarization while modulating their trafficking, ultimately influencing the duration and intensity of the pro-inflammatory phase post-MI. Thus, Metrnl plays a crucial role in cardiac repair by modulating the acute phase following myocardial infarction through its regulation of macrophage populations.

**Supplementary Information:**

The online version contains supplementary material available at https://doi.org/10.1007/s00395-026-01203-7.

## Introduction

Cardiovascular diseases are among the leading causes of death in industrialized countries [52]. Among these, myocardial infarction (MI)—an ischemic injury resulting from the occlusion of coronary vessels following acute plaque rupture—remains the leading cause of heart failure [18]. Despite improvements in therapy, the current prognosis for heart failure remains poor, with a high 12-month and 5-year all-cause mortality, mostly by cardiovascular causes [45].

MI leads to irreversible cell death of cardiomyocytes after which a reparative process is initiated to repair the infarcted area. This infarct healing process is characterized by an initial inflammation and immune cell infiltration (acute inflammatory phase) that serves to clear damaged cells and extracellular matrix (ECM) tissue, followed by an anti-inflammatory reparative phase for the resolution of inflammation, (myo)fibroblast proliferation, scar formation, and neovascularization [38, 51]. The initial inflammatory response first activates resident immune cells and endothelium, which rapidly recruit neutrophils and monocytes into the damaged tissue [17, 43, 58]. Macrophages in particular are massively recruited by the chemokine CCL2, and have a crucial role during the acute and reparative phase both by phagocyting debris, producing inflammatory cytokines and proteinases during the acute phase (days 1–3 post-MI, classic pro-inflammatory type 1 phenotype) and next, secreting mediators for inflammation resolution, ECM production, cell proliferation, myofibroblast differentiation, and angiogenesis during the reparative phase (from day 4 onwards post-MI, alternative anti-inflammatory type 2 or reparative phenotype), correlating with a T helper 1 (Th1) and Th2/Treg cytokine responses, respectively [1,2, 11, 24, 53, 55].

A prolonged pro-inflammatory phase is linked to poor clinical outcomes, including an increased risk of cardiac rupture due to impaired fibroblast activation [44]. Indeed, the magnitude of the initial inflammatory response directly influences subsequent cardiac repair and remodeling [12,13, 30, 34,35]. Therefore, achieving an appropriate balance between pro- and anti-inflammatory phases ensuring timely resolution of inflammation is a critical determinant of effective cardiac healing.

Recent studies have shown that Meteorin-like (Metrnl) drives the polarization of macrophages towards a more anti-inflammatory phenotype in the myocardium [42], adipose tissue, and skeletal muscle after tissue injury [5, 39]. Metrnl is a cytokine secreted by different cell types, including cardiomyocytes. It has been associated with innate and acquired immunity and is a regulator of the inflammatory pathways induced in alternatively activated macrophages [49,50]. We described for the first time the cardioprotective role of Metrnl against cardiac hypertrophy, through modulation of cardiac metabolism and promotion of anti-inflammatory polarization [42]. Moreover, we reported the prognostic value of METRNL circulating levels, independently predicting a worse outcome in MI patients [19]. Recently, Reboll et al*.* have described that the accumulation of macrophages expressing Metrnl in the damaged myocardium after ischemia/reperfusion injury drives a pro-angiogenic response [40]. However, the role of Metrnl in the acute phase during permanent coronary occlusion has not been studied so far.

This study investigates the role of Metrnl in facilitating the transition from the pro-inflammatory to the anti-inflammatory phase following myocardial infarction (MI)—a critical step in resolving inflammation and initiating tissue repair. Our findings demonstrate that Metrnl regulates the Osm/Reg3β axis to selectively suppress excessive inflammation while preserving the reparative functions of macrophages essential for cardiac healing.

## Materials and methods

### Animals

*Metrnl*-null mice (C57BL/6NTac-Metrnl^tm1a(KOMP)Wtsi^/WtsiH; EM:07966) were obtained from the Wellcome Trust Sanger Institute Mouse Genetics Project (Sanger MGP). Male wild-type littermates were used as controls for all experiments with *Metrnl*-null mice. All experiments were performed in accordance with European Community Council directive 86/609/EEC and were approved by the Animal Experimentation Ethics Committee of the University of Barcelona, Sant Pau Institute for Biomedical Investigation and Local Government Animal Care and Use Committee (11,671).

### AAV vectors

AAV9-null and AAV9-Metrnl were generated by the CBATEG (Barcelona) using a dual-triple plasmid co-transfection procedure followed by polyethylene glycol (PEG) precipitation and purification through CsCl_2_ gradient centrifugations. Mice were intraperitoneally (i.p.) injected at day 7 of age with AAV9-null (CT) or AAV9-Metrnl at a dose of 1 × 10^13^ viral genome particles per animal, as previously described [42].

### Regional myocardial infarction

MI was induced by coronary artery ligation. To this end, mice were anesthetized with a mixture of O_2_/isoflurane (2%) in an induction chamber. A midline cervical incision was performed to expose the trachea, and each mouse was intubated with an endotracheal tube connected to a small animal ventilator. Anesthesia was maintained during the surgical procedure by isoflurane inhalation (1.5–2.5%). Body temperature was monitored with a rectal probe and maintained at 37 °C using a warming pad. Following a left-side thoracotomy between the 3rd and 4th ribs, the heart was exposed, and the left anterior descending (LAD) coronary artery was ligated just proximal to its main branching point with a 7-0 silk suture (Assut Europe, Margliano dei Marsi, AQ, IT). The infarcted area was visualized after ligation by development of a pale color in the distal myocardium. Sham procedures were identical with exception of the actual tying of the suture. The thorax was closed with 6-0 vicryl sutures (Ethicon, Raritan, NJ, USA). Analgesia was administrated subcutaneously (buprenorphine, 0.1 mg/kg) prior to surgery and continued postoperatively for 3 days via drinking water (buprenorphine, 0.03 mg/mL). Mice were euthanized 4 and 21 days after MI or sham surgery using a cardioplegic solution (68.4 mM NaCl, 59 mM KCl, 11.1 mM glucose, 1.9 mM NaHCO3, 29.3 mM 2,3-butanedione monoxime, and 1000 U heparin), after previous anesthesia [31].

### Echocardiography measurements

Echocardiography analysis of cardiac function was conducted at baseline, 2 and 4 days post-MI or sham operation. Mice were lightly anesthetized with isoflurane (1–1.5%) and placed on a heating pad (37 °C). Trans-thoracic echocardiography was performed using a 55 MHz linear array transducer interfaced with a Visual Sonics Vevo2100 High-Resolution In Vivo Imaging System (FUJIFILM VisualSonics, Toronto, CA). Standard parasternal long- and short-axis views were obtained in B and M modes, and functional parameters were measured using Simpson’s method. All images were captured by a single operator with expertise in mouse echocardiography who was blinded to genotype.

After completing the measurements, the hearts were arrested in diastole with cardioplegic solution, excised, the atria were removed, and the ventricles were weighed. Subsequently, the hearts were fixed for 24 h in 4% paraformaldehyde dissolved in PBS and cryopreserved in 30% sucrose overnight at 4 ºC. The hearts were then embedded and frozen in Tissue-Tek O.C.T. compound (Sakura) and stored at − 80 °C. For RNA analysis, the hearts were divided into the remote zone, border peri-ischemic zone, and ischemic zone, and snap frozen for later RNA analysis. The infarct area was defined as a pale, semi-opaque region surrounding the ligation point; the border zone as a 1 mm-wide perimeter adjacent to the infarct; and the remote zone as a distal myocardial region.

### Histochemistry and immunostaining

For histological examination, tissues were serially cryosectioned at a thickness of 10 μm, and stained with Picrosirius red. All sections were blindly examined and photographed using a Leica M80 (MDG33) stereoscope. Assessment of the infarcted area as percentage of myocardium volume was performed on serial sections from apex to base, as previously described [6].

Cell immune infiltration was evaluated by immunofluorescence staining using primary antibodies against neutrophils, Ly6B (Ref. ab53457, 1/50 Abcam, Cambridge, UK) and macrophages, F4/80 (Ref. #30,325, 1/50, Cell Signaling, Danvers, MA, United States) and secondary antibodies conjugated with A647 and A594, respectively (1 µg/mL, Jackson ImmunoResearch, Cambridge, UK). Samples were observed in confocal microscope Leica SP5 AOBS (Leica, Biosystems, Nussloch, Germany) and Zeiss LSM 980 AiryScan 2 (Zeiss, Oberkochen, Germany). Images were quantified using QuPath software.

To determine vascular density at the ischemic and border zones, mouse heart sections were incubated with a primary antibody against CD31 (Ref. ab62169; 0.25 µg/mL, Abcam, Cambridge, UK). A secondary antibody conjugated with Alexa488 (1 µg/mL; Jackson ImmunoResearch, Cambridge, UK) was used for detection. Images were taken from at least 8 randomly selected fields (4 ischemic zones and 4 border zones) and analyzed using image analysis software (Fiji, NIH). The results were expressed as a percentage of the mean CD31-positive area per area of tissue surface.

### Flow cytometry

To determine monocyte activation, whole blood samples were collected 4 days post-MI and stained with Ly6G-FITC (Ref. 127,605 Clone 1A8; BioLegend, San Diego, CA, United States), Ly6C-APC (Ref. 130–123-796 Clone 1G7.G10, Milteny, Germany), CD45-APC/Cy7 (Ref. 157,203 Clone S18009F, BioLegend), F4/80-BV711 (Ref. 123,147 Clone BM8, BioLegend), CD11b-BV785 (Ref. 101,243 Clone M1/70, BioLegend), CCR2-PE (Ref 568,093 Clone Y15-488. rMAb, BD Biosciences, Franklin Lakes, NJ, United States), and CCR5-PE/Cy7 (Ref. 107,018 Clone HM-CCR5, BioLegend). For the spleen, splenocytes were isolated 4 days post-MI and stained with Ly6G-FITC, Ly6C-APC, CD45-APC/Cy7, F4/80-BV711, CD11b-BV785, CD86-PE/Cy7 (Ref. 105,116 Clone PO3, BioLegend), and Arg1-PE (Ref. 12–3697-80 Clone A1exF5, Invitrogen, Carlsbad, CA, United States). Samples were acquired in an LSR Fortessa cytometer (BD Biosciences) and data were analyzed with the FlowJo software (v10.8.1, BD, Franklin Lakes, NJ, United States). The gate strategy we followed is detailed in Supplemental Figure S1.

### RNA extraction and quantitative RT-PCR

Total RNA was extracted using Tripure (Roche, Indianapolis, IN, USA). RT was performed using a High-Capacity RNA-to-cDNA kit (Applied Biosystems, Foster City, CA, USA) and 0.5 µg RNA in a total reaction volume of 20 μl. Quantitative RT-PCR was conducted in duplicate for increased accuracy. Taqman Gene Expression Assays (Thermo Fisher Scientific, Waltham, MA USA) were used; each 20-μl reaction mixture contained 1 μl cDNA, 10 μl TaqMan Universal PCR Master Mix (Thermo Fisher Scientific), 250 nM probes, and 900 nM primers from the Assays-on-Demand Gene Expression Assay Mix or the Assays-by-Design Gene Expression Assay Mix (Thermo Fisher Scientific). Total reaction mixture was completed with deionized MiliQ water. Each sample was run in duplicate and the mean value was used to calculate the mRNA expression of the gene of interest and the housekeeping reference gene (Cyclophilin A, PPIA). The mRNA level of the gene of interest in each sample was normalized to that of the reference control using the comparative (2^−ΔCT^) method.

### Single-nucleus RNA (snRNA) sequencing data analysis

Preparation and quality control of libraries were performed as follows. Library concentration was assessed using Qubit 2.0 fluorometric quantification. Insert size verification was conducted to evaluate fragment length distribution, and the final effective library concentration was determined by quantitative PCR (qPCR) to ensure optimal cluster density during sequencing. Qualified single-cell libraries were subsequently pooled according to their precisely measured effective concentrations and target data outputs and sequenced on Illumina high-throughput platforms. Raw sequencing files (FASTQ format) were processed on a per-sample basis using the Cell Ranger software suite (10 × Genomics) with default parameters. Reads were aligned to the Mus musculus GRCm38 reference genome using the embedded STAR (Spliced Transcripts Alignment to a Reference) algorithm, an ultrafast RNA-seq aligner [15]. For quantification, Cell Ranger performed cell barcode filtering, unique molecular identifier (UMI) counting, and gene expression matrix generation, yielding high-quality cell-by-gene count tables. Following matrix generation, downstream analysis was conducted using the Bioconductor ecosystem and the Seurat framework. Single-nucleus data integration was performed using canonical correlation analysis (CCA) or mutual nearest neighbors (MNN) anchoring to correct for batch effects [9]. Dimensionality reduction and visualization were subsequently applied, and Uniform Manifold Approximation and Projection (UMAP) was used to preserve global data structure and define cell clusters [7]. Data were log-normalized, and highly variable features were identified using a variance-stabilizing transformation (VST) method. Each cell cluster was annotated according to its cell type using the CellMarker 2.0 [23] and PanglaoDB [20] databases, which harbor well-characterized cell type-specific markers. Subclustering of macrophages was performed using MACanalyzeR, following the protocols and vignettes published by the authors [36]. Data are deposited in Gene Expression Omnibus (GEO) under the accession number GSE335342.

### Analysis of METRNL and OSM circulating levels in mouse

Blood samples were obtained 4 days after MI or sham operation and centrifuged for 5 min at 10,000 g in a Z-gels tubes (Sarstedt, Nümbrecht, Germany). Plasma was collected and stored at -20 ºC until analyzed.

METRNL and OSM circulating levels were determined using the spectrophotometry immunoassay mouse METRNL ELISA kit (Ref. DY6679) and mouse Oncostatin M (OSM) Quantikine ELISA Kit (Ref, MSM00, R&D Systems), respectively. Absorbance was measured in a Multiskan FC spectrophotometer (Thermo Fisher Scientific).

### Western-blot analysis

Whole-tissue lysates were homogenized in a MILLIPLEX MAP Lysis buffer for Multiplexing (Merck, Sigma-Aldrich, Darmstadt, Germany), also containing 1 mM phenylmethylsulfonyl fluoride (PMSF) and a multiple protease inhibitor mix (Roche Diagnostics, Basel, Switzerland). After protein quantification with the Pierce BCA Protein Assay Kit (Thermofisher Scientific), 20 µg of denatured protein extracts were loaded and separated in 12% SDS-PAGE gels and transferred into Immobilon-P membranes (Millipore). Western-blot analyses were performed using primary antibodies against OSM (NBP3-16,686, Novus Biologicals; 1:1000).

### Neonatal cardiomyocyte (NCM) cell culture

Rat NCMs were obtained as previously described [37]. Briefly, hearts were digested with a collagenase solution (Collagenase Type I; Life Technologies, Ltd., Paisley, UK) followed by differential plating. NCMs were stimulated with the hypertrophic agent phenylephrine (PE, 10 µM; Sigma), OSM (40 ng/ml, R&D systems), or CoCl2 (500 µM, Sigma) for 24 h. To mimic hypoxic conditions, NCMs were cultured in 1% Oxygen, 5% CO2 and 94% N2 v/v for up to 24 h.

Recombinant adenovirus expressing the murine Metrnl cDNA was constructed as previously described [42] (Ad5-CMV-Metrnl; CBATEG, Barcelona). NCMs were infected with Metrnl adenoviral vector and the AdCMV-null control vector at 10 IFU/cell for 24 h in serum-free medium.

### RAW 264.7 cell culture

Murine male RAW 264.7 macrophages were grown in RPMI-1640 medium supplemented with 10% heat-inactivated FBS (iFBS) and 1% penicillin/streptomycin (P/S) (Life Technologies) at 37 °C in a humidified 5% CO_2_ incubator. Cells between passages 15 and 20 were used for the experiments shown. For gene expression assays, macrophages were plated at a concentration of 2.5 × 10^5^ cells·mL^−1^. Once they reached 80% confluence, cells were serum-starved and kept in RPMI medium supplemented with 0.05% bovine serum albumin (BSA) for 12 h. Then, macrophages were either classically activated to a pro-inflammatory phenotype with 60 ng·mL^−1^ (LPS; Sigma-Aldrich) or alternatively activated to an anti-inflammatory/reparative phenotype with 40 ng·mL^−1^ IL-4 (PeproTech) for 12 h. Thereafter, cells were used for chemotaxis assays (see below) or were treated with 10 nM of METRNL (R&D systems) or REG3β (MyBiosource) for 12 h. Cells were then harvested and RNA was extracted as described above.

### Bone marrow-derived macrophages (BMDM) cell culture

BMDM protocol was adapted from two previously described protocols [10]. Tibia and femur of 8-week-old wt and Metrnl-KO mice were collected, and bone marrow was extracted by centrifugation with DMEM. Cells were differentiated for 7 days, with medium changes at days 2 and 5, in DMEM supplemented with 10% iFBS, 10% L929-cell conditioned medium and 1% P/S in a humidified 5% CO2 incubator at 37 °C. L929-cell conditioned medium was obtained from a 10-day cell culture, maintained in DMEM supplemented with 10% iFBS. After differentiation period, cells were serum starved (1% iFBS) for 12 h and activated into pro-inflammatory or anti-inflammatory macrophages with LPS (60 ng·mL-1) or IL-4 (40 ng·mL-1), respectively, for an additional 12 h.

### Chemotaxis assay

Macrophage chemotaxis was assessed using Costar Transwell chambers on 24-well plates (Corning Incorporated, NY, USA). Macrophages activated with LPS or IL-4 were trypsinized and suspended in serum-free RPMI or DMEM for RAW 264.7, respectively. The bottom wells of the chemotaxis plates were filled with conditioned media from NCMs overexpressing Adnull or AdMetrnl. Pro- and anti-inflammatory-activated macrophage suspensions (2 × 10^5^ cells in 200 μL) were placed in the upper wells where they were separated from the lower wells by polycarbonate membranes with 8.0 μm pores. Plates were then incubated at 37 °C for 20 h. Afterward, media from the upper wells were removed and chambers were rinsed with 1 × PBS. Cells were fixed with a 4% formaldehyde solution for 15 min, washed with PBS, stained with hematoxylin for 30 min at room temperature, and finally washed in water. Non-migrating cells were gently scrapped from the upper side of the membranes and migrating macrophages on the lower side were visualized on an inverted microscope. Cell counts were determined using ImageJ 2.0 software.

### Human samples

Patients with STEMI who were admitted to a tertiary university center within a primary percutaneous coronary intervention (PCI) network were included in the study. MI with ST-segment elevation (STEMI) was diagnosed and managed according to the contemporary guidelines. The protocol was approved by the institutional ethics committee (references EO-11-061, PI-24-027) and patients or their representatives provided written informed consent to use their samples for scientific purposes. Samples from the control population were requested to the Germans Trias i Pujol Biobank collection IGTP-HUGTP registered in the National Registry of ISCIII (B.0000643) to determine the levels in the general population.

### ELISA analysis of human samples

Blood samples were obtained by venipuncture 12 or 16 h after symptoms onset for METRNL and OSM assessment, respectively. Plasma was obtained by centrifugation and stored at -80ºC until assayed. Circulating levels of METRNL were measured by Human Meteorin-like/METRNL DuoSet ELISA (R&D Systems, United States; reference DY786) and Oncostatin M (OSM) ELISA (Elabscience, China; reference E-EL-H2247) according to the manufacturer’s protocols. Absorbance was measured using Varioskan Flash (Thermo Fisher Scientific, Waltha, MA, United States).

### Statistics

Cell culture experiments were conducted on at least three independent NCM isolations. The number of animals used were indicated in the Figure legends. Results are presented as mean ± SD. Data were analyzed by T test, One- and Two-way ANOVA, or Mann–Whitney tests, followed by post hoc tests as appropriate, using the GraphPad Prism software v8.0.1 (GraphPad Software Inc., San Diego, CA, USA). A p value less than 0.05 was considered statistically significant.

## Results

### MI increases cardiac and circulating levels of Metrnl

**Fig. 1:**
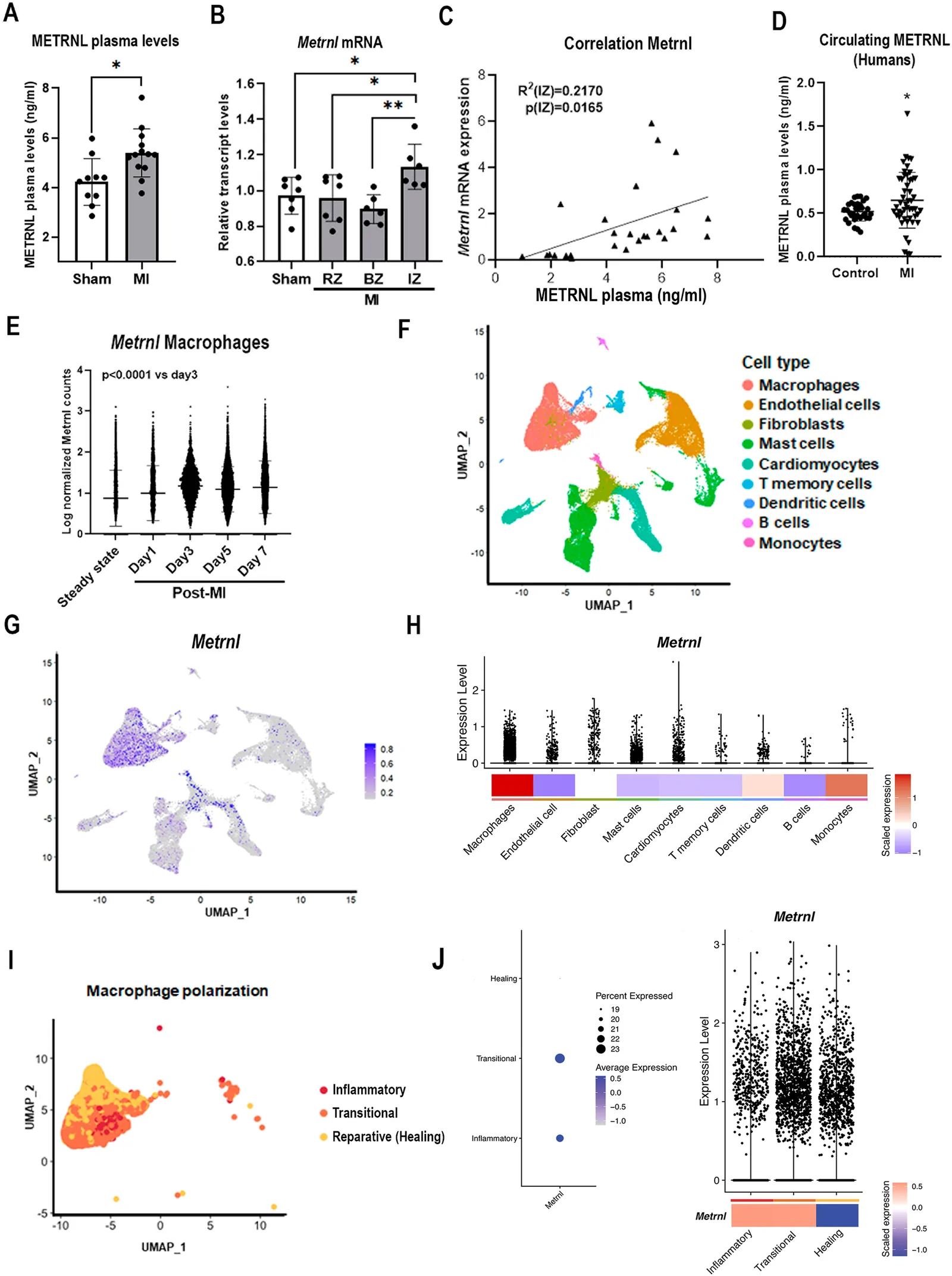
Metrnl is expressed and secreted during the acute phase post-MI. 17-week-old wt mice were subjected to sham operation (Sham, white bars) or permanent coronary artery ligation (MI, grey bars) and samples were analyzed 4 days after surgery. **A** Circulating METRNL protein levels; *n* = 10–13 mice/group. **B** *Metrnl* expression levels in the myocardium of sham or MI mice in the remote zone (RZ), border zone (BZ), and ischemic zone (IZ); *n* = 6–7 mice/group. **C** Correlation between cardiac *Metrnl* expression levels in the IZ and METRNL circulating levels in wt, *Metrnl*^*−/−*^*,* and AAV9Metrnl mice 4 days post-MI. **D** Circulating levels of METRNL in control individuals (mean age 60 ± 2.63) and MI patients (mean age 60 ± 11.70). **E** Violin-plot representation of normalized Metrnl counts from single-cell RNA-seq (sc-RNAseq) macrophage sub-cluster at days 0, 1, 3, 5, and 7 post-MI. **F**–**J** Single-nucleus RNA-seq (snRNA-seq) of wt, *Metrnl⁻/⁻*, and *Metrnl⁻/⁻* + AAV9-Metrnl mouse hearts at day 4 post-MI (n = 2 mice/group). **F** Two-dimensional UMAP visualization of cardiac nuclei identifying nine major cell types. **G** Two-dimensional UMAP showing expression levels of *Metrnl* and (**H**) violin-plot and heatmap displaying the expression pattern of *Metrnl* across the different cell types identified in this dataset. **I** Two-dimensional UMAP showing subclustering analysis of macrophages from snRNA-seq of mouse hearts at day 4 post-MI. Macrophage polarization visualization identifies three subclusters. Each point represents a single cell; macrophage types are color-coded (inflammatory-red, transitional-orange, and reparative/healing-yellow). **J** Violin-plot and heatmap showing the expression pattern of *Metrnl* across the three macrophage subtypes. Results are expressed as mean ± SD. Data were analyzed by paired (A, B), unpaired t test (D), Pearson test (C), or one-way ANOVA, followed by Dunnett’s post hoc test for multiple comparisons against the day 3 group (E). ^*^p < 0.05, ^**^p < 0.01 compared to corresponding control [Sham mice, RZ, Steady state or Controls]

To study the involvement of the secreted protein Metrnl during the initial phase post-MI, we subjected mice to permanent occlusion of the LAD coronary artery, and we analyzed the cardiac response 4 days post-MI, an appropriate time point to study macrophage populations in the myocardium [33]. At this time point, the acute inflammatory phase ends, and the reparative anti-inflammatory phase begins, providing a temporal overlap to study the switch between both phases. First, we characterized Metrnl levels in wild-type animals, finding a significant increase in METRNL circulating levels 4 days post-MI compared to sham animals (Fig. 1A). Moreover, when we analyzed the myocardium in detail, we found that Metrnl transcript levels were significantly increased in the ischemic zone (IZ) in wild-type animals compared to sham animals, but not in the border (BZ) or remote zones (RZ) (Fig. 1B). In addition, the expression levels of *Metrnl* in IZ were significantly higher compared to RZ and BZ. Interestingly, METRNL circulating levels positively correlate with *Metrnl* mRNA expression in the IZ (Fig. 1C) but not with the *Metrnl* expression in the BZ (*p* = 0.5026) or RZ (*p* = 0.1551), suggesting that the increased Metrnl production in the IZ is the source of the elevated METRNL circulating levels.

We also analyzed the circulating METRNL levels in control individuals (*n* = 32; mean age ± SD: 60 ± 2.63; 40% females) and patients suffering from MI (*n* = 49; mean age 60 ± 11.70; 15% females) (Fig. 1D), finding significantly higher circulating levels of METRNL in MI patients compared to control individuals.

Altogether, these results indicate that permanent MI damage is characterized by increased circulating and cardiac Metrnl levels.

Although cardiomyocytes produce Metrnl [42], myeloid cells are considered the main source of Metrnl in the myocardium post-MI [40]. Thus, we next analyzed the immune cell transcriptome dynamics of Metrnl at a single-cell level in the mouse heart after permanent coronary artery ligation, analyzing longitudinal scRNA-seq data at steady state, 1-, 3-, 5-, and 7-days post-MI deposited by Jung et al*.* [25] to the Gene Expression Omnibus (GEO) repository under the accession number GSE163129. The total clustering results of immune cells are presented in Fig. 1E, showing similar cluster populations and cell fractions as the original analysis [25]. A total of 33,251 cells captured after quality control filtering (6,509 cells at steady state; 6,801 cells at day 1 post-MI; 4,368 cells at day 3 post-MI; 7,291 cells at day 5 post-MI; 8,282 cells at day 7 post-MI) were integrated, clustered, and visualized in uniform manifold approximation and projection (UMAP) plots using the Seurat R package [48]. To annotate cell clusters, the SingleR package [3] was used to assess the expression of well-known cell-type-specific markers, defining a total of 12 broad cell clusters within CD45^+^ cells. The most abundant immune cell population was macrophages (65.01% of total CD45^+^ cells), with highly expressed prototypical macrophage genes, such as Csf1r, Cd68, and Adgre1. Non-macrophage cell populations included the same as the ones showed in the reference paper: DC), Xcr1^+^ DC, migratory DC, monocytes, neutrophils, B cells, T cells, group 2 innate lymphoid cells (ILC2), natural killer (NK) cells, plasma cells, and mast cells (Supplemental Figure S2A). The representation of the temporal clustering post-MI is shown in Supplemental Figure S2A. Briefly, in the steady state, macrophages represented the largest cell population in the mouse hearts, which drastically dropped at day 1 post-MI but then gradually recovered from day 3 post-MI. By contrast, neutrophils peaked at day 1 and then decreased rapidly. Monocytes were recruited in the early phase of MI (days 1 and 3) and decreased later (days 5 and 7), while DCs were at steady minority at days 3 and 5. Lymphocytes (T cells, B cells, NK cells, and ILC2 cells) were decreased from day 1 onwards.

We next analyzed the expression levels of *Metrnl* in the clustering post-MI, and we found that among the immune cells, neutrophils, macrophages, and monocytes expressed the highest levels of *Metrnl* (Supplemental Figure S2B). Specifically, the characterization of cell dynamics showed that *Metrnl* expression was restricted to neutrophils and monocytes at day 1 post-MI and mainly present in monocytes and macrophages from day 3 to day 7.

Since the largest population of immune cells expressing Metrnl in the heart are macrophages, we performed a subclustering analysis of macrophages, obtaining 7 subclusters (Supplemental Figure S2C). We defined our monocyte/macrophage populations taking as reference the cluster identification made by Jung et al., obtaining indeed very similar clustering results. The study of population dynamics of the different subclusters showed that most of the macrophages disappeared at day 1 post-MI, when circulating monocytes (Ly6C^+^) are recruited to the myocardium. From day 3 to 7, all the different macrophage populations are progressively represented in the cardiac tissue. When analyzing the temporal dynamics of Metrnl expression in the total macrophage population, we observed a peak at day 3 post-MI, followed by a significant decline thereafter. This pattern supports the relevance of Metrnl during the inflammatory phase post-MI (Fig. 1E and Supplemental Figure S2D). Notably, the peak at day 3 reflects an increased mean expression level compared to other time points, driven by a relatively small subset of cells exhibiting elevated expression levels.

Since this previous analysis was restricted to immune cells, we next performed single-nucleus RNA sequencing (snRNA-seq) of the myocardium 4 days post-MI to comprehensively assess Metrnl expression across all cardiac cell types in our mice (Fig. 1F-J). The two-dimensional UMAP visualization of cardiac nuclei identified nine major cell types within the myocardium: macrophages, endothelial cells, fibroblasts, mast cells, cardiomyocytes, T memory cells, dendritic cells, B cells, and monocytes (Fig. 1F). Next, we mapped *Metrnl* expression within these cell populations (Fig. 1G, H). Our analysis revealed that, although immune cells represent a significant source of Metrnl, its expression is not limited to this compartment. Notably, several non-immune cell populations, including cardiomyocytes, also expressed Metrnl, corroborating our previous observations [42]. Despite this broader distribution, macrophages emerged as the predominant cell type expressing the highest levels of Metrnl in the post-MI myocardium. To further investigate the relationship between *Metrnl* expression and macrophage functional states, we performed a subclustering analysis of the macrophage compartment (Fig. 1I). Based on established transcriptional signatures [36], macrophages were categorized into inflammatory, transitional, and reparative (healing) subsets (Fig. 1J). Strikingly, inflammatory macrophages exhibited the highest levels of *Metrnl* expression, followed by transitional macrophages, whereas reparative macrophages displayed the lowest expression levels.

These findings indicate that *Metrnl* expression is dynamically regulated during macrophage polarization and is particularly enriched during the early pro-inflammatory phase following MI.

### Cardiac healing post-MI requires Metrnl

**Fig. 2:**
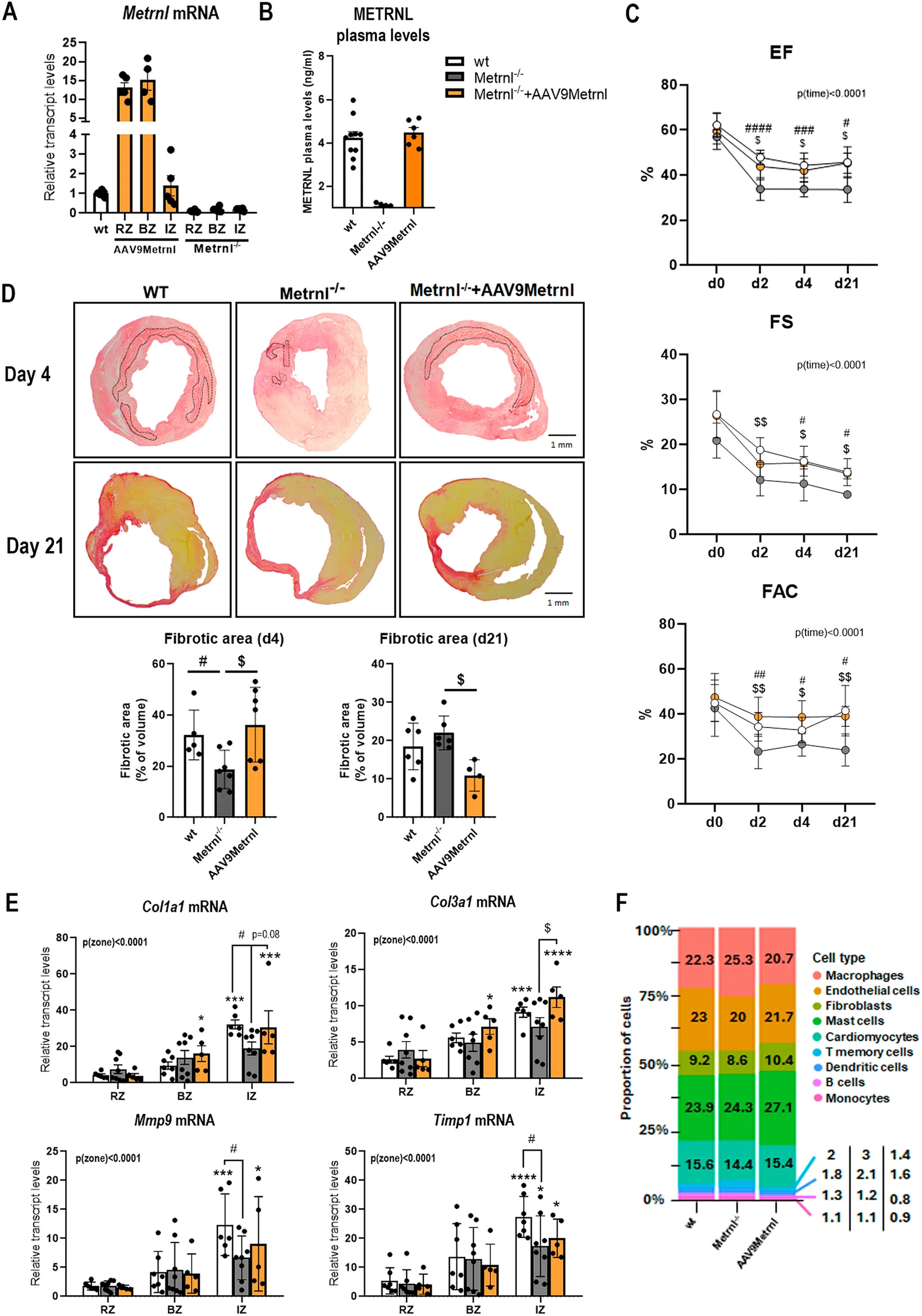
Metrnl prevents cardiac dysfunction and enhances remodeling after MI. 17-week-old wt (white bars), *Metrnl*^*−/*−^ mice injected i.p with AAV9-null (*Metrnl*^*−/−*^*,* grey bars) or with AAV9 carrying Metrnl (AAV9Metrnl, orange bars) were subjected to permanent coronary artery ligation for 4 and 21 days. The myocardium of MI mice was divided in three zones: remote zone (RZ), border zone (BZ), and ischemic zone (IZ) **(A)** *Metrnl* expression levels in mouse heart post-MI; *n* = 4–8 mice/group **(B)** Circulating METRNL protein levels post-MI; *n* = 6–10 mice/group **(C)** Echocardiographic data from wt, Metrnl^−/−^ and AAV9Metrnl mice after 2, 4, and 21 days post-MI induction. EF: ejection fraction, FS: fractional shortening, FAC: Fractional area change. *n* = 12–15 mice/group. **D** Quantification of fibrotic area in histological heart sections stained with Picrosirius red 4 and 21 days post-MI. Scale bar 1 mm. Lines show the fibrotic tissue. Results are expressed as a percentage of total myocardium; *n* = 4–7 mice/group. **E** Gene expression of fibrotic markers *Col3a1, Col1a1, Mmp9, and Timp1* in RZ, BZ, and IZ at day 4 post-MI; *n* = 5–8 mice/ group. **F** Single-nucleus RNA-seq (snRNA-seq) of wt, *Metrnl⁻/⁻*, and *Metrnl⁻/⁻* + AAV9-Metrnl mouse hearts at day 4 post-MI (*n* = 2 mice/group). Proportion of cell types from the two-dimensional UMAP visualization of cardiac nuclei. Results are expressed as mean ± SD. Data were analyzed by t test, one-way ANOVA, or two-way ANOVA. ^*^p < 0.05, ^**^p < 0.01, ^***^p < 0.001, ^****^p < 0.0001 compared with corresponding RZ (D, E); ^#^*p* < 0.05, ^##^*p* < 0.01 compared with corresponding wt mice; ^$^*p* < 0.05 compared with corresponding *Metrnl*^*−/−*^ mice

Since Metrnl appeared to be of relevance during the initial phase post-MI, we next studied the impact of Metrnl ablation (*Metrnl*^−/−^) together with the rescue of Metrnl in the myocardium of *Metrnl*^−/−^ mice using cardiotropic adeno-associated viral vectors expressing Metrnl (*Metrnl*^−/−^ + AAV9-Metrnl) 4 days post-MI (Fig. 2).

We have previously demonstrated the preferential cardiac targeting of adeno-associated viral vector AAV9 in vivo and reported the AAV9-mediated expression of Metrnl in the heart [42]. In our MI model, we confirmed the absence of Metrnl expression in the myocardium of *Metrnl*^−/−^ mice, in which Metrnl was restored following AAV9-Metrnl injection (Fig. 2A). We found that Metrnl was overexpressed in the RZ and BZ and restored to wt levels in the IZ of the myocardium of *Metrnl*^−/−^ mice injected with AAV9-Metrnl. Moreover, circulating METRNL levels in *Metrnl⁻/⁻* mice treated with AAV9-Metrnl returned to values comparable to those observed in wt mice at baseline (Fig. 2B), highlighting the contribution of the myocardium to the circulating METRNL pool in the context of a global Metrnl deficiency, particularly given that METRNL is produced by multiple tissues beyond the myocardium.

Next, we analyzed the cardiac function of wt, *Metrnl*^−/−^ and *Metrnl*^−/−^ + AAV9-Metrnl mice 2, 4, and 21 days post-MI using echocardiography (Fig. 2C). We found that the left-ventricular ejection fraction (EF), the fractional shortening (FS) and the fractional area change (FAC) were significantly decreased in *Metrnl*^*−/−*^ mice compared with wt animals at all time points post-MI, indicating that Metrnl ablation leads to a greater cardiac dysfunction of the myocardium post-MI than wt mice. By contrast, in *Metrnl*^−/−^ + AAV9-Metrnl mice, the EF, FS, and FAC were significantly increased compared with *Metrnl*^*−/−*^ mice, reaching the levels of wt mice post-MI. Overall, these results indicate that overexpression of Metrnl specifically in the myocardium is sufficient to rescue cardiac function in mice lacking Metrnl.

We also assessed the extent of the fibrotic area at 4 and 21 days post-MI by staining fibrotic tissue with Picrosirius red (Fig. 2D). At 4 days post-MI, the fibrotic area was significantly reduced in *Metrnl⁻/⁻* mice compared to both wt and *Metrnl⁻/⁻* + AAV9-Metrnl animals, indicating impaired cardiac healing in the absence of Metrnl. Notably, overexpression of Metrnl restored the fibrotic area to levels comparable to WT mice. In contrast, at 21 days post-MI, the fibrotic area tended to be larger in *Metrnl⁻/⁻* mice compared to wt animals and was significantly reduced in *Metrnl⁻/⁻* + AAV9-Metrnl mice relative to *Metrnl⁻/⁻* mice. These findings indicate that the magnitude of the pro-inflammatory phase and its contribution to the initiation of fibrotic scar formation are critical determinants of final scar size.

In addition, we analyzed the transcript levels of fibrosis marker genes, such as collagen 3 (*Col3a1*), collagen 1 (*Col1a1*), matrix metalloproteinase-9 (*Mmp9*), and tissue inhibitor of metalloproteinase-1 (*Timp1*) 4 days post-MI. We found that all genes were significantly increased in the IZ compared with RZ of wt and *Metrnl*^−/−^ + AAV9-Metrnl mice, but not in *Metrnl*^*−/−*^ mice (Fig. 2E). Moreover, the expression levels of *Col1a1, Mmp9, and Timp1* in the IZ were significantly reduced in *Metrnl*^*−/−*^ mice compared to wt mice, and Metrnl overexpression increased the expression levels of *Col3a1* and tended to increase *Col1a1* expression (p = 0.08) when compared to *Metrnl*^−/−^ mice (Fig. 2E). Finally, the percentage survival of *Metrnl*^*−/−*^ mice, but not *Metrnl*^−/−^ + AAV9-Metrnl mice, tends to be reduced in line with the cardiac alterations observed (Supplemental Figure S3).

Consistent with these findings, analysis of cell-type populations in our snRNA-seq data revealed a reduced proportion of fibroblasts and an increased proportion of macrophages in *Metrnl*^*−/−*^ mice. In contrast, *Metrnl*^*−/−*^ mice treated with AAV9-Metrnl exhibited increased proportion of fibroblasts, along with a reduction in macrophages (Fig. 2F).

In summary, Metrnl abrogation reduces myocardial healing, leading to cardiac dysfunction after MI. Furthermore, cardiac Metrnl overexpression restores the wt response to MI in *Metrnl*^*−/−*^ mice.

### Metrnl is necessary for efficient activation of reparative macrophages to the damaged heart after MI

**Fig. 3:**
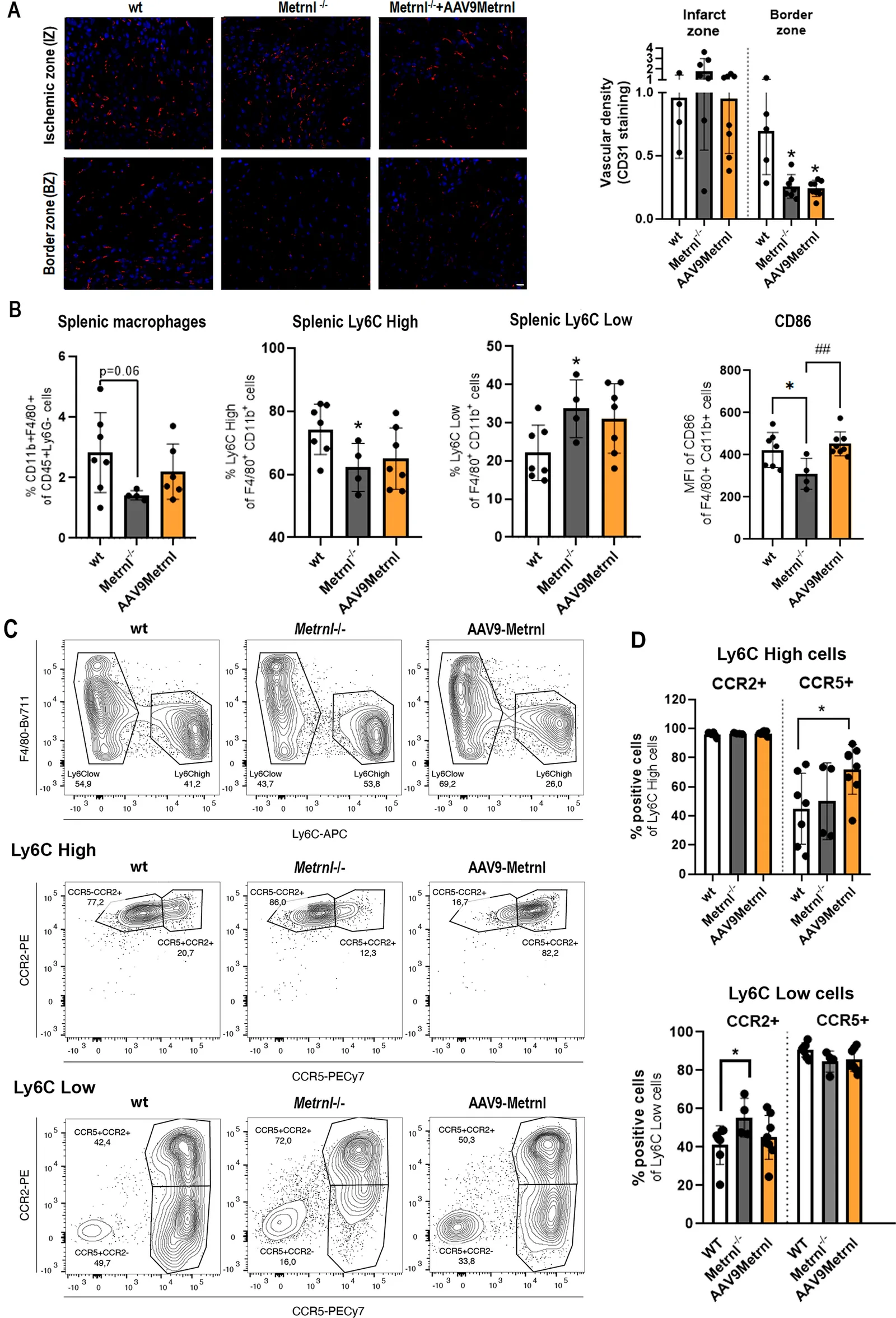
Metrnl deficiency promotes recruitment of inflammatory cells after MI. 17-week-old wt (white bars), *Metrnl*^*−/−*^ mice injected i.p with AAV9-null (*Metrnl*^*−/−*^*,* grey bars) or with AAV9 carrying Metrnl (AAV9Metrnl, orange bars) were subjected to permanent coronary artery ligation for 4 days. **A** Determination of vascular density by immunofluorescence staining of CD31 protein (red) in IZ and BZ. Scale bar 20 µm. DAPI was used to stain nuclei (blue). Results are expressed as the percentage of the area occupied by the CD31 signal; *n* = 4–8 mice/group. **B** Histograms representing the percentage of macrophages, Ly6C High, Ly6C Low and expression of CD86 (MFI) in spleen 4 days post-MI; *n* = 4–8 mice. **C** Representative graph of CCR2^+^ or CCR5^+^ Ly6C populations in blood 4 days post-MI. **D** Histograms representing the percentage of macrophages CCR2^+^ or CCR5^+^ Ly6C in blood 4 days post-MI; *n* = 4–8 mice/group. Data are expressed as mean ± SD. Data were analyzed by t test and one-way ANOVA as appropriated. ^*^p < 0.05 compared with wt mice; ^#^*p* < 0.05, ^##^*p* < 0.01 compared with *Metrnl*^*−/−*^ mice

Given that the proposed mechanism of cardiac repair promotion by Metrnl is neovascularization [40], we next analyzed vascular density by performing anti-CD31 immunostaining of the myocardium (Fig. 3A). We found no significant differences in the vascular density of the IZ between groups. However, we found a significant decrease in vascularization in the BZ of both *Metrnl*^−/−^ and *Metrnl*^−/−^ + AAV9-Metrnl mice compared to wt animals. In accordance, the proportion of endothelial cells in our sn-RNAseq was similar in both *Metrnl*^−/−^ and *Metrnl*^−/−^ + AAV9-Metrnl mice (Fig. 2F). These data suggest that the protective effect of AAV9-Metrnl on cardiac healing at this early stage occurs independently of any pro-angiogenic influence.

Several studies have shown that reparative macrophages critically determine the repair of infarcted adult murine heart by regulating fibroblast activation and scar formation [33, 44]. Considering that mice lacking Metrnl develop an altered response to healing that is restored when overexpressing cardiac Metrnl, and that macrophages are one of the main sources of Metrnl post-MI (Fig. 1), we next studied the macrophage response in wt, *Metrnl*^−/−^ and *Metrnl*^−/−^ + AAV9-Metrnl mice 4 days post-MI.

We performed flow cytometry analysis of the populations of leukocytes in blood and spleen, to analyze the circulating populations and the main source of recruited monocytes post-MI. The presented flow cytometry data expressed as % reflect shifts in cell population composition. In spleen, the macrophage (CD45^+^Ly6G^−^F4/80^+^CD11b^+^) population was reduced (p = 0.06) in *Metrnl*^−/−^ mice compared to wt animals but not in *Metrnl*^−/−^ + AAV9-Metrnl mice (Fig. 3B). Moreover, the subpopulation of Ly6C^high^ macrophages was significantly reduced and the Ly6C^low^ macrophages significantly increased in the spleen of *Metrnl*^−/−^ mice compared to wt mice. The activation marker CD86 was decreased in F4/80^+^CD11b^+^ macrophages in the *Metrnl*^*−/−*^ group compared to the wt group, whereas it was recovered in *Metrnl*^*−/−*^ + AAV9-Metrnl. These data may indicate that activated Ly6C^high^ monocytes are mobilized from the spleen to the blood circulation after MI especially in the absence of Metrnl, whereas only less activated Ly6C^low^ monocytes are kept in the spleen of *Metrnl*^−/−^ mice.

In blood, we observed no differences in Ly6C^high^CCR2^+^ monocyte populations between genotypes (Fig. 3C, D). However, we detected a significant increase in CCR5 expression by Ly6C^high^ monocytes in *Metrnl*^−/−^ + AAV9-Metrnl mice compared to wt mice. By contrast, Ly6C^low^CCR2^+^ monocyte populations were unchanged in *Metrnl*^−/−^ + AAV9-Metrnl mice but significantly increased in *Metrnl*^−/−^ mice compared to wt mice, indicating a predominant inflammatory monocyte profile in blood of mice lacking Metrnl. No significant changes in Ly6C^low^CCR5^+^ monocyte populations were observed between genotypes.

Flow cytometry analysis of wt and *Metrnl*^−/−^ mice at baseline was also performed to discard baseline alterations in leukocyte populations in the spleen or blood finding no genotype differences (Supplemental Figure S4A).

**Fig. 4:**
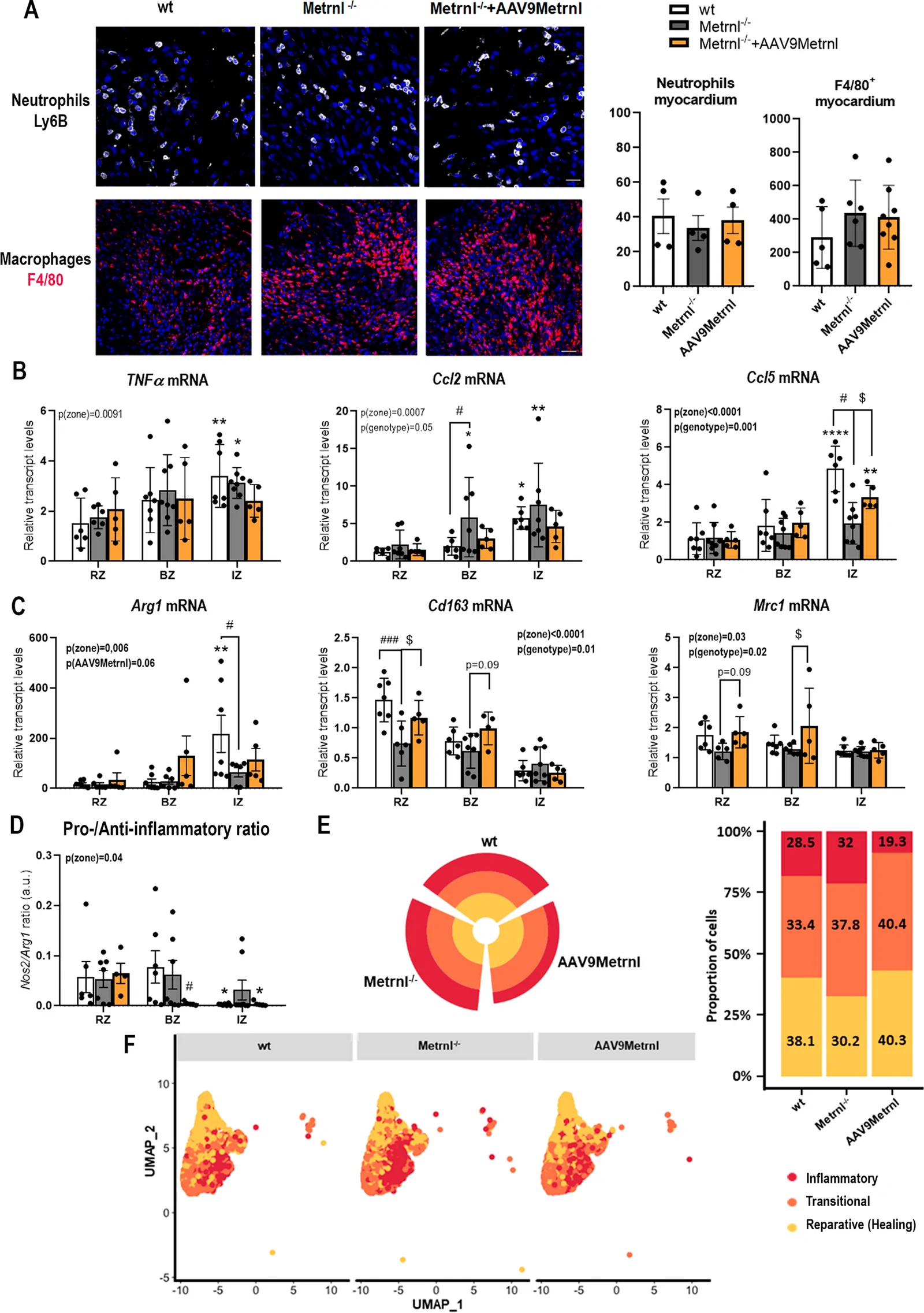
Metrnl^−/−^ mice develop worse inflammatory response in the myocardium after MI. 17-weeks old wt (white bars), Metrnl^−/−^ mice injected i.p. with AAV9-null (*Metrnl*^*−/−*^*,* grey bars) or with AAV9 carrying Metrnl (AAV9Metrnl, orange bars) were subjected to permanent coronary artery ligation for 4 days. The myocardium of MI mice was divided in three zones: remote zone (RZ), border zone (BZ), and ischemic zone (IZ).** A** Quantification of immune cell infiltration in the IZ 4 days post-MI by immunofluorescence staining of macrophages (F4/80, red) and neutrophils (Ly6B, white) in heart. DAPI was used to stain nuclei (blue). Scale bar: 20 µm. Results are expressed as number of cells/field; *n* = 4–8 mice/group. **B** mRNA expression levels of inflammatory marker *TNFα* and ligands of chemokine receptors *Ccl2 and Ccl5; n* = 5–8 mice/group. **C** mRNA expression levels of anti-inflammatory markers *Arg1, Cd163, Mrc1*; *n* = 5–8 mice/group. **D** Pro-inflammatory/anti-inflammatory macrophage ratio, expressed as iNos/Arg1 expression level ratio; *n* = 5–8 mice/group. **E–F** Single-nucleus RNA-seq (snRNA-seq) of wt, *Metrnl⁻/⁻,* and *Metrnl⁻/⁻* + AAV9-Metrnl mouse hearts at day 4 post-MI (*n* = 2 mice/group). Two-dimensional UMAP showing subclustering analysis of macrophages from snRNA-seq of mouse hearts at day 4 post-MI. Macrophage polarization visualization identifies three subclusters. Each point represents a single cell; macrophage types are color-coded (inflammatory-red, transitional-orange, and reparative/healing-yellow). **E** Radar plot and bar plot showing the proportion of macrophages at each polarization stage per condition. **F** UMAPs showing subclustering for each condition. Results are expressed as mean ± SD. Data were analyzed by t test and two-way ANOVA as appropriated. ^*^p < 0.05; ^**^p < 0.01 compared with corresponding RZ; ^#^*p* < 0.05, ^##^*p* < 0.01, ^###^*p* < 0.001 compared with corresponding wt mice; ^$^*p* < 0.05 compared with corresponding *Metrnl*^*−/−*^ mice

Next, we analyzed the presence of neutrophils (Ly6B) and macrophages (F4/80) in the myocardium of wt, *Metrnl*^−/−^ and *Metrnl*^−/−^ + AAV9-Metrnl mice 4 days post-MI (Fig. 4). As expected, no differences were observed in the number of neutrophils in the myocardium at this time point. Moreover, immunofluorescence staining of total F4/80^+^ macrophages revealed no changes in the number of total macrophages in the infarcted area of the myocardium in any condition (Fig. 4A).

To determine the activation state of infiltrating macrophages, we analyzed the gene expression of the pro-inflammatory macrophage marker tumor necrosis factor (*Tnf),* and the C–C motif chemokine ligand 2 (*Ccl2*) (pro-inflammatory ligand recruiting CCR2^+^ cells)*,* the C–C motif chemokine ligand 5 (*Ccl5* or *Rantes)* (recruiting anti-inflammatory CCR5^+^ cells), and the reparative macrophage markers: Arginase-1 (*Arg1)*, scavenger receptor-163 (*Cd163)*, and mannose receptor C-type 1 (*Mrc1)* in the myocardium of these animals (Fig. 4B, C). We found that the transcript levels of *Tnf* and *Ccl2* were significantly increased in the IZ of the myocardium compared with the RZ in wt and in *Metrnl*^−/−^ mice, but not in *Metrnl*^−/−^ + AAV9-Metrnl mice (Fig. 4B). Global genotype analysis showed a significant effect for *Ccl2* (p = 0.05), with a specific significant increase in the BZ of *Metrnl*^−/−^ mice compared to wt mice. Regarding *Ccl5*, we found a significant increase in the IZ compared to the RZ and BZ of wt and *Metrnl*^−/−^ + AAV9-Metrnl mice, but not in *Metrnl*^−/−^ mice. When comparing genotypes, the expression levels of *Ccl5* were significantly lower in *Metrnl*^−/−^ mice. These data indicate that *Metrnl*^−/−^ mice would recruit more pro-inflammatory CCR2^+^ monocytes to the myocardium, while *Metrnl*^−/−^ + AAV9-Metrnl would instead recruit more anti-inflammatory CCR5^+^ monocytes, in accordance with the increase observed in circulating CCR2^+^ monocytes and CCR5^+^ monocytes, respectively (Fig. 3).

Regarding the expression of reparative/anti-inflammatory macrophage markers, we observed that the reparative marker *Arg1* was upregulated only in IZ compared to BZ in wt mice (Fig. 4C). When we compared genotypes, we found that the transcript levels of *Arg1* were significantly reduced in *Metrnl*^−/−^ mice compared to wt animals in IZ. Moreover, the mRNA levels of other reparative markers *Cd163* and *Mrc1* were overall reduced in the RZ of *Metrnl*^−/−^ mice compared to wt mice. By contrast, the expression levels of *Cd163* and *Mrc1* were increased in the RZ and BZ of *Metrnl*^−/−^ + AAV9-Metrnl mice compared to *Metrnl*^−/−^ mice. In both cases, there was also a global genotype effect. Finally, and more importantly, the pro-inflammatory versus anti-inflammatory ratio indicating inflammatory macrophage activation, estimated based on the iNOS/Arg1 ratio [41], was significantly increased in the IZ of the heart of *Metrnl*^−/−^ mice compared to wt and *Metrnl*^−/−^ + AAV9-Metrnl mice (Fig. 4D). Moreover, in *Metrnl*^−/−^ + AAV9-Metrnl mice, the pro-inflammatory/anti-inflammatory ratio was further significantly reduced in BZ compared to wt mice. Altogether, these results show a predominant inflammatory population in the myocardium of *Metrnl*^−/−^ mice and a predominant reparative/anti-inflammatory population in *Metrnl*^−/−^ mice overexpressing cardiac Metrnl.

Our snRNA-seq data further substantiated these observations (Fig. 4E, F). Specifically, macrophage polarization analysis revealed a marked shift in macrophage composition depending on Metrnl levels. In *Metrnl⁻/⁻* mice, the macrophage compartment was predominantly composed of pro-inflammatory and transitional subsets, indicating a sustained pro-inflammatory state. In contrast, mice overexpressing Metrnl displayed a higher proportion of reparative/anti-inflammatory (healing) macrophages, accompanied by a reduction in pro-inflammatory macrophages.

In parallel, we overexpressed Metrnl in wt mice using the same strategy of AAV9-Metrnl and we analyzed the animals 4 days post-MI (Supplemental Figure S4B-E). As expected, we found a significant increase of Metrnl mRNA levels in wt mice overexpressing Metrnl (Supplemental Figure S4B) in all areas of the heart post-MI, although this upregulation was not accompanied by changes in cardiac function at this early point (Supplemental Fig. 4C). However, we found that the infarcted area tends to be increased (p = 0.08) in mice overexpressing Metrnl compared to wt animals. Moreover, the number of total (F4/80^+^) and reparative/anti-inflammatory (CD260^+^) macrophages in the myocardium was also increased in mice overexpressing Metrnl compared to wt animals (Supplemental Fig. 4D). In accordance, the expression levels of *Arg1* were significantly increased and the pro-inflammatory versus anti-inflammatory ratio was decreased in wt + AAV9-Metrnl compared to wt mice (Supplemental Fig. 4E).

Collectively, these data indicate that Metrnl plays a critical role in modulating macrophage phenotype following myocardial infarction. Elevated Metrnl levels appear to promote the transition from a pro-inflammatory to a reparative environment, thereby facilitating resolution of inflammation and progression toward tissue repair. Conversely, the absence of Metrnl skews macrophages toward a more inflammatory profile, which may impair proper healing and exacerbate cardiac injury.

### Oncostatin M is regulated by Metrnl after MI

**Fig. 5:**
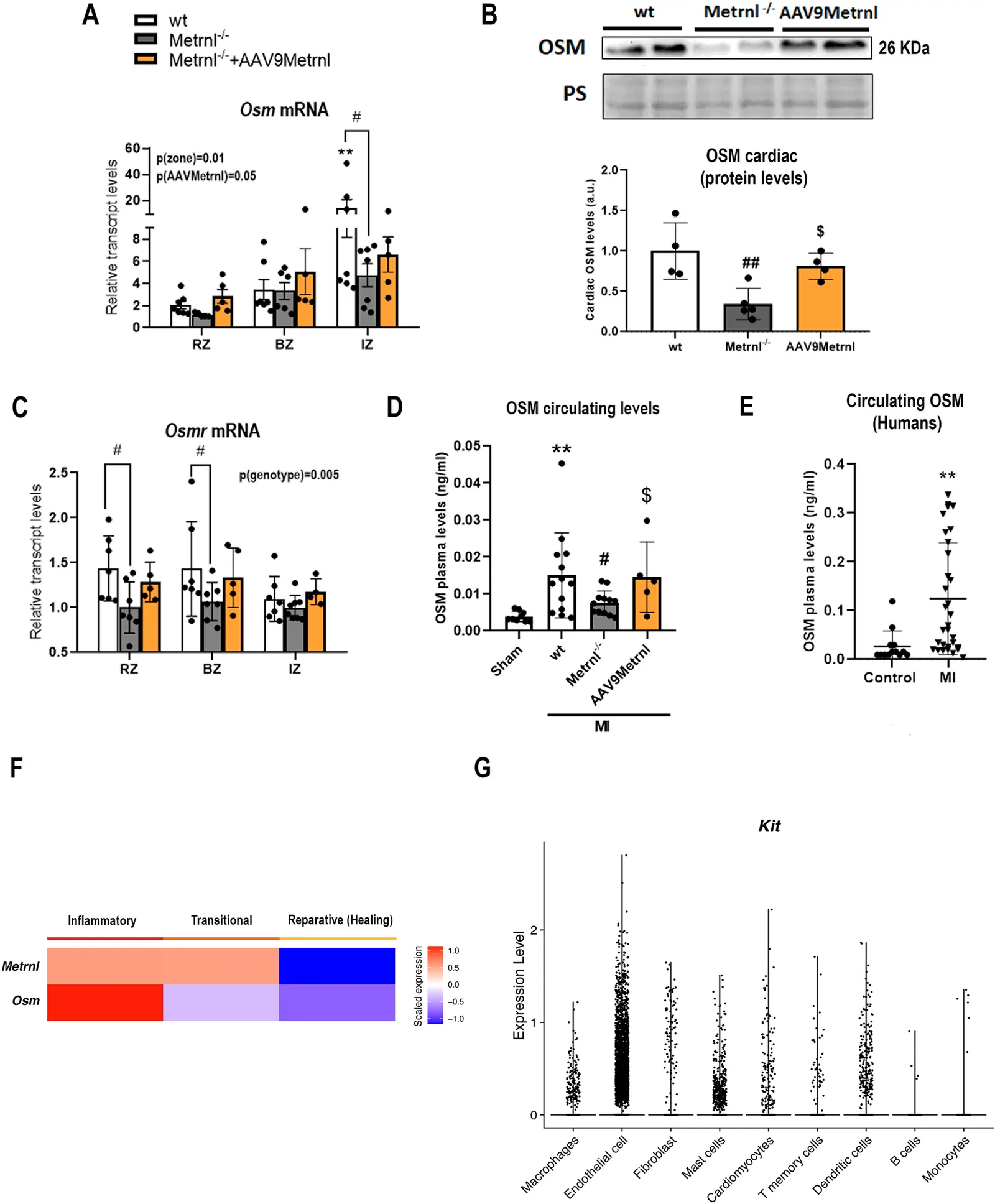
Oncostatin M (Osm) levels are regulated by Metrnl in mice post-MI. 17-week-old wt (white bars), *Metrnl*^*−/−*^ mice injected i.p with AAV9-null (*Metrnl*^*−/−*^*,* grey bars) or with AAV9 carrying Metrnl (AAV9Metrnl, orange bars) were subjected to permanent coronary artery ligation for 4 days. The myocardium of MI mice was divided in three zones: remote zone (RZ), border zone (BZ), and ischemic zone (IZ). **A** mRNA levels of *Osm* in RZ, BZ, and IZ; n = 5–8 mice/group. **B** Representative images and quantification of OSM protein levels in IZ 4 days post-MI induction by western blot analysis. Ponceau S were used as loading control; n = 4–5 mice/group. **C** mRNA levels of *Osmr* in RZ, BZ, and IZ; n = 5–8 mice/group. **D** OSM circulating protein levels; n = 5–13 mice/group. **E** Plasmatic OSM protein levels in control individuals (Control) (59 ± 6.63) and MI patients (MI) (70 ± 10.2). **F, G** Single-nucleus RNA-seq (snRNA-seq) of wt, *Metrnl⁻/⁻,* and *Metrnl⁻/⁻* + AAV9-Metrnl mouse hearts at day 4 post-MI (n = 2 mice/group). **F** Heatmap displaying the expression pattern of *Metrnl* and *Osm* across the three macrophage subtypes. **G** Violin-plot of *Kit (*c-kit) expression across the different cell types. Results are expressed as mean ± SD. Data were analyzed by t test and two-way ANOVA. ^*^p < 0.05, ^**^p < 0.01 compared with corresponding RZ; ^#^*p* < 0.05, ^##^*p* < 0.01 compared with wt group; $, *P* < 0.05 compared with *Metrnl*^*−/−*^ group

Osm is an interleukin 6 (IL6)-type cytokine produced by neutrophils and inflammatory macrophages during the acute phase post-MI and has a critical role in determining the extent of the pro-inflammatory phase [27, 33]. We studied the Osm profile in our experimental setting 4 days post-MI. As expected, we found that the mRNA expression levels of Osm in the myocardium were significantly increased in IZ compared to RZ only in wt animals (Fig. 5A). Instead, although inflammatory macrophages are more actively recruited in *Metrnl*^−/−^ mice, the transcript levels of *Osm* were significantly lower in the IZ of *Metrnl*^−/−^ mice compared to wt mice. By contrast, the mRNA expression levels of *Osm* were significantly upregulated in all regions of the myocardium of *Metrnl*^−/−^ + AAV9-Metrnl mice compared to *Metrnl*^−/−^ mice (pAAVMetrnl = 0.05). In wt + AAV9-Metrnl mice, we also found a significant increase in the mRNA expression levels of *Osm* compared to wt mice (p = 0.01) (Supplemental Fig. 4E). We further confirmed the transcriptomics data with protein analysis of OSM, performing western blot analysis of IZ tissue (Fig. 5B). We found that OSM protein levels were significantly reduced in the myocardium of *Metrnl*^−/−^ mice, whereas their protein levels were significantly recovered after Metrnl overexpression in *Metrnl*^−/−^ + AAV9-Metrnl mice. Accordingly, the expression levels of the well-known Osm receptor (*Osmr),* highly expressed in cardiomyocytes [27], were significantly reduced in the RZ and BZ of *Metrnl*^−/−^ mice compared to wt mice, but not in *Metrnl*^−/−^ + AAV9-Metrnl mice (Fig. 5C).

The changes in Osm mRNA and protein levels observed in the myocardium were accompanied by parallel changes in OSM circulating levels (Fig. 5D). We observed increased OSM levels in serum of wt mice subjected to MI compared to sham mice, while the increase was blunted in *Metrnl*^−/−^ mice subjected to MI. Finally, OSM circulating levels were significantly increased in *Metrnl*^−/−^ + AAV9-Metrnl mice to the same levels as wt mice.

Next, we analyzed the OSM circulating levels in control individuals (n = 14; 59 ± 6.63 years; 43% female) and patients suffering from MI (n = 30; 70 ± 10.21 years, 53% female) (Fig. 5E). We found that the circulating levels of OSM were significantly higher in MI patients compared to control individuals.

Consistent with the observed pattern for Metrnl, our snRNA-seq analysis revealed that *Osm* expression followed a similar distribution across macrophage subsets. Specifically, *Osm* expression was highest in inflammatory macrophages, intermediate in transitional macrophages, and lowest in reparative (healing) macrophages, although its overall expression levels were lower compared to *Metrnl* (Fig. 5F). Interestingly, the Metrnl receptor c-Kit [40] was also detected in macrophages, in addition to other cardiac cell types, suggesting that macrophages are likely responsive to Metrnl signaling (Fig. 5G). Together, these findings suggest that Metrnl may act upstream to regulate *Osm* expression, supporting a potential mechanistic link between these factors in controlling macrophage function during cardiac injury and repair.

### Metrnl induces *Osm* expression and secretion specifically in inflammatory activated macrophages

**Fig. 6:**
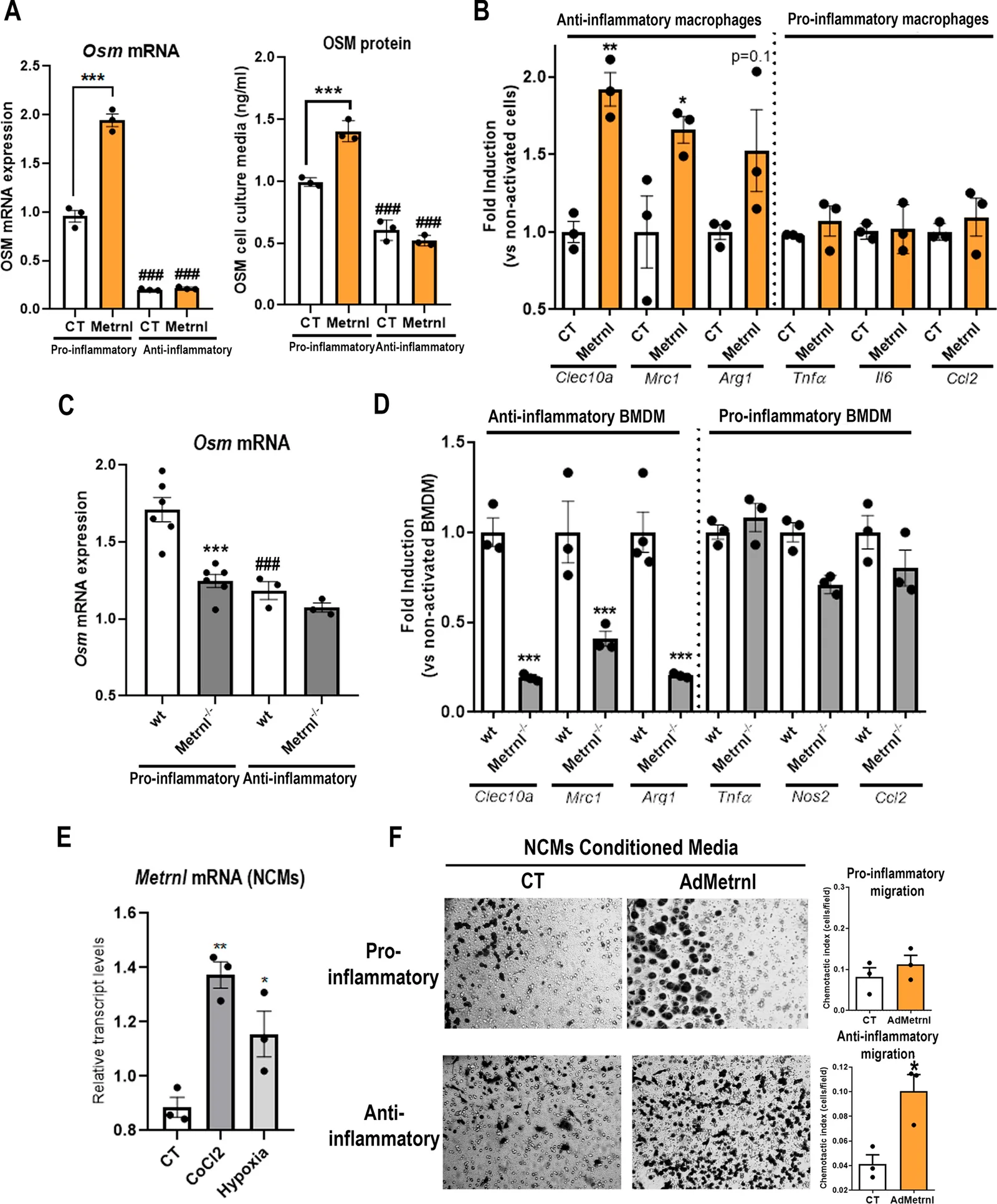
Metrnl regulates Osm in macrophages in vitro. **A** *Osm* mRNA and OSM protein levels in cell culture media of pro- and anti-inflammatory-activated RAW264.7 macrophages treated with PBS (control, white bars) or Metrnl (orange bars) for 24 h. **B** Expression levels of anti-inflammatory (*Clec10a*, *Mrc1, Arg1*) and pro-inflammatory (*Tnf*, *Il6, Ccl2*) markers in activated RAW264.7 macrophages treated with PBS (control, white bars) or Metrnl (orange bars) for 12 h. Results are expressed as fold induction versus their corresponding non-activated cells. **C** Expression levels of *Osm* in activated bone marrow-derived macrophages (BMDM) obtained from wt (white bars) and *Metrnl*^*−/−*^ mice (grey bars). **D** Expression levels of anti-inflammatory (*Clec10a*, *Mrc1, Arg1*) and pro-inflammatory (*Tnf*, *Nos2, Ccl2*) markers in activated BMDM obtained from wt (white bars) and *Metrnl*^*−/−*^ mice (grey bars). Results are expressed as fold induction versus their corresponding non-activated cells and set at 1 for graphical representation. **E** Expression levels of *Metrnl* in primary neonatal cardiomyocytes in culture (NCMs) subjected to the hypoxia mimetic CoCl_2_ or hypoxic conditions (1% Oxygen, 5% CO2 and 94% N2 v/v). **F** Migration assay showing the number of pro-inflammatory and anti-inflammatory macrophages migrating in response to cell culture media of NCMs previously infected with null adenovirus (CT) or Metrnl adenovirus (AdMetrnl). Cell culture experiments were conducted in at least three independent cardiomyocyte isolations. Data are represented as mean ± SD. ^*^p < 0.05, ^**^p < 0.01, and ***p < 0.001 versus control cells or wt mice; ###*p* < 0.001 versus pro-inflammatory-activated macrophages. Two-tailed Student’s t test and one-way ANOVA were used

To elucidate whether the observed Metrnl-mediated in vivo induction of Osm production post-MI is due to a direct effect on macrophages, we performed in vitro studies using RAW 264.7 macrophages. After inducing macrophage polarization into pro-inflammatory (Lipopolysaccharide, LPS) or anti-inflammatory/reparative (Interleukin-4, IL4) states, we confirmed their activation by assessing the expression levels of typical pro-inflammatory markers (*Tnf, Il6* and *Ccl2*) and anti-inflammatory markers (*Clec10a, Mrc1* and *Arg1*) (Supplemental Figure S5A)*.* We found a significant increased expression of *Tnf, Il6,* and *Ccl2*, or *Clec10a, Mrc1,* and *Arg1,* when macrophages were treated with LPS or IL4, respectively, thus confirming pro-inflammatory and anti-inflammatory/reparative macrophage polarizations. Next, we analyzed the expression levels of *Osm* and *Metrnl* in this in vitro experimental system, showing that only pro-inflammatory-activated macrophages produced both *Metrnl* and *Osm* (Supplemental Figure S5B). Next, we treated pro- and anti-inflammatory-activated macrophages with recombinant METRNL and we found that Metrnl treatment significantly increased both the mRNA expression levels and the secreted protein levels of OSM, but only in pro-inflammatory activated macrophages, not in anti-inflammatory activated macrophages. This indicates that Metrnl further upregulates Osm production only in pro-inflammatory macrophages (Fig. 6A). Interestingly, treatment with Metrnl significantly upregulated the anti-inflammatory markers *Clec 10a, Mrc1*, and *Arg1* but not pro-inflammatory markers *Tnf, Il6*, and *Ccl2* (Fig. 6B). These data indicate that Osm regulation by Metrnl is specific, since key inflammatory mediators typically associated with pro-inflammatory macrophages such as Ccl2, Il6, and Tnfα remain unchanged upon Metrnl treatment.

To further confirm the Metrnl-dependent regulation of Osm, we performed studies using primary bone marrow-derived macrophages BMDM cell cultures from wt and *Metrnl*^−/−^ mice, either pro-inflammatory or anti-inflammatory activated (Fig. 6C, D). We found that the expression levels of *Osm* were higher in pro-inflammatory versus anti-inflammatory BMDM from wt mice. Moreover, *Osm* levels were significantly lower in pro-inflammatory but not in anti-inflammatory BMDM of *Metrnl*^−/−^ mice when compared to wt animals (Fig. 6C). Interestingly, anti-inflammatory BMDM from *Metrnl*^−/−^ mice showed a significant reduction in the expression levels of the anti-inflammatory markers *Clec 10a, Mrc1,* and *Arg1* compared to BMDM from wt mice (Fig. 6D). However, pro-inflammatory BMDM from *Metrnl*^−/−^ mice responded similarly to wt, with no changes in *Tnf, Nos2* or *Ccl2.* We confirmed the presence of c-Kit in BMDMs (Supplemental Figure S5C). These results further confirm the involvement of Metrnl in Osm production by pro-inflammatory macrophages and in anti-inflammatory macrophage polarization.

We have previously shown that cardiomyocytes are also a target and source of Metrnl [42]. To analyze the Metrnl production by cardiomyocytes under hypoxia-like condition, we treated primary cultures of NCMs with cobalt chloride (CoCl_2_), a well-known hypoxia mimetic mediator, or hypoxic conditions (1% Oxygen, 5% CO2 and 94% N2 v/v) for up to 24 h. We found that after both hypoxic conditions, the expression levels of *Metrnl* were significantly increased (Fig. 6E), indicating that cardiomyocytes can contribute to the increased Metrnl levels observed post-MI.

At last, to study our in vivo situation in which macrophages are recruited to the post-MI damaged myocardium after cardiac Metrnl overexpression, we analyzed the chemoattractant properties of the conditioned media from NCMs overexpressing Metrnl (AdMetrnl). We confirmed the increased mRNA expression of *Metrnl* and its release into the cell culture media (Supplemental Figure S5D). We found that cardiomyocytes overexpressing Metrnl preferentially induce the migration of anti-inflammatory-activated macrophages (Fig. 6F).

We conclude that Metrnl produced by cardiomyocytes and immune cells within the infarcted area specifically induces Osm expression in pro-inflammatory macrophages. In parallel, Metrnl promotes a shift toward an anti-inflammatory/reparative macrophage phenotype and supports the preferential recruitment of these cells. These effects are likely mediated through direct interaction with its receptor, c-Kit, expressed on macrophages. However, c-Kit knock-down studies are needed to definitely conclude that Metrnl acts through this receptor in macrophages.

### Reg3β inhibits pro-inflammatory macrophage differentiation and determines the duration of the inflammatory phase

**Fig. 7:**
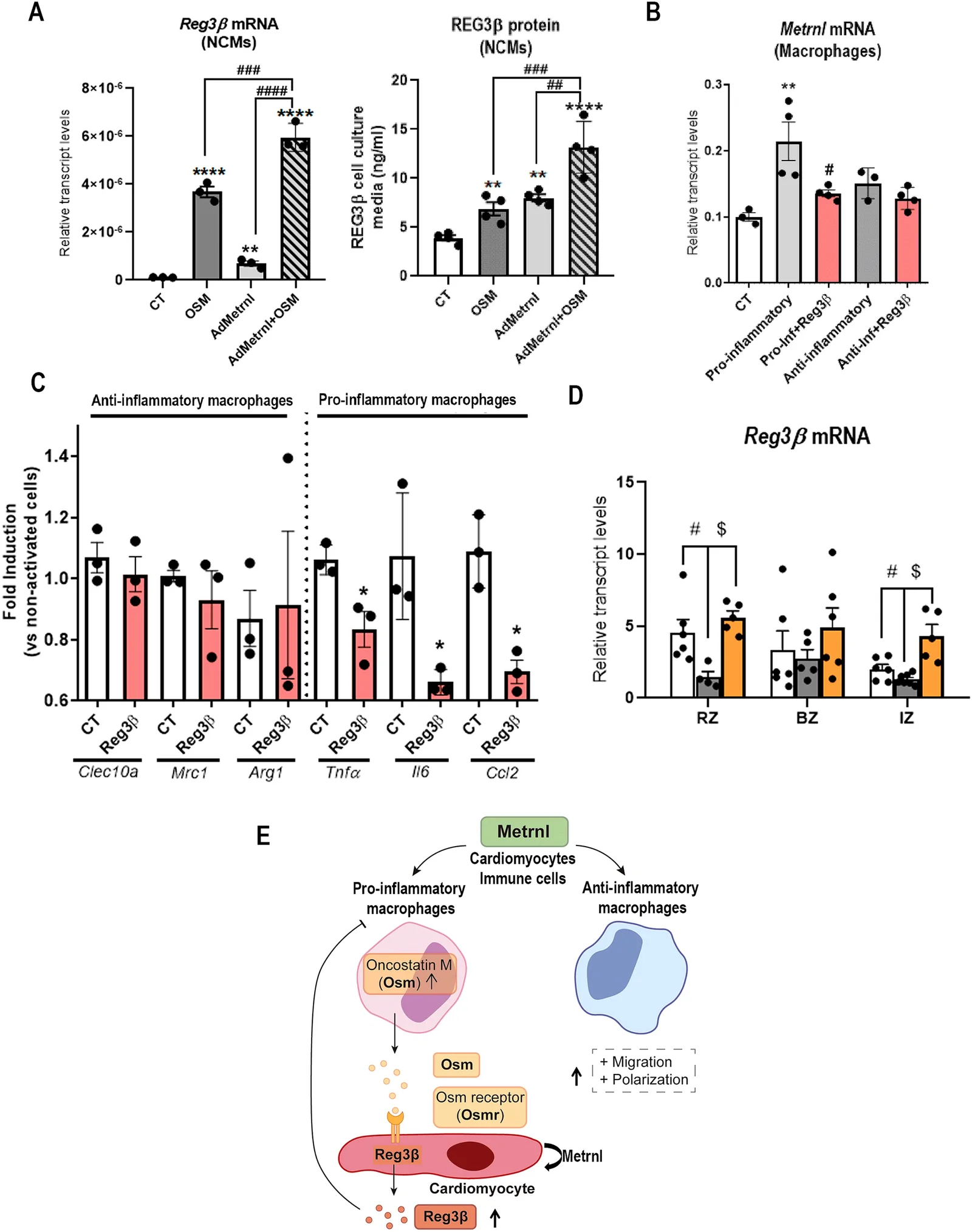
Reg3β secreted by cardiomyocytes and regulated by the Metrnl–Osm axis inhibits Metrnl. **A** mRNA transcript levels of *Reg3β* and REG3β protein levels in cell culture media of neonatal cardiomyocytes (NCMs) previously infected with null adenovirus (CT) or Metrnl adenovirus (AdMetrnl) and treated with/without Osm for 24 h. **B** *Metrnl* mRNA levels in RAW264.7 macrophages (pro-inflammatory or anti-inflammatory-activated) in the presence or absence of recombinant Reg3β protein for 12 h during the activation process. **C** Expression levels of anti-inflammatory (*Clec10a*, *Mrc1, Arg1*) and pro-inflammatory (*Tnf*, *Il6, Ccl2*) markers in activated RAW264.7 macrophages treated with PBS (control, white bars) or Reg3β (pink bars) for 12 h. Results are expressed as fold induction versus their corresponding non-activated cells and set at 1 for graphical representation. **D** *Reg3β* expression levels in the myocardium of wt (white bars), *Metrnl*^*−/−*^ mice injected i.p. with AAV9-null (*Metrnl*^*−/−*^, grey bars) or with AAV9 carrying Metrnl (AAV9Metrnl, orange bars) subjected to permanent coronary artery ligation for 4 days. Cell culture experiments were conducted on at least three independent cardiomyocyte isolations. Data are represented as mean ± SD. ^*^p < 0.05; ^**^p < 0.01; ^***^p < 0.001; ^****^p < 0.0001 compared with corresponding control cells or wt mice; ^#^*p* < 0.05; ^##^*p* < 0.01; ^###^*p* < 0.001; ^####^*p* < 0.0001 as indicated [in A and D] or pro-inflammatory macrophages [in B]. Data were analyzed by t test and one-way ANOVA. **E** Schematic representation of the proposed mechanism of action of Metrnl on macrophages post-MI

Reg3β is a cardiomyocyte-secreted protein regulated by Osm, involved in the recruitment of macrophages into the infarcted area [33]. To analyze the impact of Metrnl over Reg3β production in our in vitro system, we treated NCMs overexpressing Metrnl with Osm. As expected, Osm treatment increased *Reg3β* expression levels and REG3β secreted protein levels in the cell supernatant (Fig. 7A). Metrnl overexpression (AdMetrnl) had the same effects on Reg3β as OSM treatment, indicating that endogenously produced Metrnl in NCMs acts in an autocrine manner and regulates Reg3β production. Moreover, the combination of AdMetrnl together with Osm treatment further increased Reg3β expression and secretion into the cell culture media, indicative of a synergistic effect.

Then, to determine whether Reg3β, in addition of being a potent chemoattractant [33], also affects macrophage differentiation, we treated pro-inflammatory and anti-inflammatory macrophages with Reg3β. We observed that Reg3β addition during the pro-inflammatory differentiation process significantly reduced the expression levels of *Metrnl* to those of undifferentiated monocytes, whereas Reg3β addition did not affect the Metrnl mRNA production of anti-inflammatory macrophages (Fig. 7B). Moreover, Reg3β treatment did not affect anti-inflammatory macrophage differentiation but reduced the pro-inflammatory markers *Tnf, Il6* and *Ccl2* (Fig. 7C). These data indicate that Reg3β inhibits Metrnl production by pro-inflammatory macrophages and interferes in pro-inflammatory macrophage polarization but does not affect anti-inflammatory macrophage polarization.

Finally, we analyzed the expression levels of Reg3β in the myocardium of wt, *Metrnl*^−/−^ and *Metrnl*^−/−^ + AAV9-Metrnl mice 4 days post-MI (Fig. 7D). We found that the transcript levels of Reg3β were significantly reduced in the myocardium of *Metrnl*^−/−^ mice compared to wt mice but were restored in *Metrnl*^−/−^ + AAV9-Metrnl mice.

## Discussion

In this study, we demonstrate that Metrnl plays a crucial role in macrophage polarization and cardiac healing post-MI, regulating Osm production by pro-inflammatory macrophages and favoring an anti-inflammatory macrophage polarization in the myocardium. Moreover, we describe a negative feedback loop in which Reg3β secreted by cardiomyocytes and regulated by the Metrnl-Osm axis inhibits Metrnl production and pro-inflammatory macrophage polarization. Altogether, it makes Metrnl a critical regulator of the inflammatory response post-MI, regulating the extent and duration of the acute inflammatory phase and promoting in turn the start of the reparative phase post-MI (Fig. 7E).

The transition from the pro-inflammatory to the anti-inflammatory phase is a crucial step in resolving inflammation and initiating tissue repair, as pro-inflammatory macrophages must first fulfill their functions before shifting to the reparative phenotype [2, 28]. Our findings reveal that mice lacking Metrnl exhibit defective tissue repair, marked by a prolonged and insufficiently suppressed pro-inflammatory phase. This leads to sustained tissue damage, impaired scar formation, and contractile dysfunction, ultimately promoting chamber dilation 4 days post-MI. These results align with studies showing that selective depletion of anti-inflammatory macrophages and inactivation of Osm and Reg3β post-MI results in impaired cardiac function and disturbed scar formation [33, 44]. Furthermore, our data indicate that mice genetically deficient in Metrnl fail to transition toward an anti-inflammatory phenotype, consistent with the previous findings in skeletal muscle repair [5, 29] and cardiac hypertrophy models [42]. However, when Metrnl was specifically restored in the myocardium, we observed an increase in reparative macrophages in the ischemic area and proper scar formation, in line with prior reports demonstrating that reparative/anti-inflammatory macrophages regulate fibroblast activation [33, 44].

The requirement of monocytes and macrophages for cardiac healing in neonatal and adult hearts after myocardial injury has been demonstrated in a few studies [4, 33, 44]. After MI, activated monocytes are mobilized from the spleen and bone marrow into the circulation to be recruited to the border and infarct zones [22, 47]. Indeed, we observed a deleterious response to permanent MI in Metrnl-deficient mice, which was associated with a predominant pro-inflammatory-like phenotype but no alterations in the total number of macrophages accumulated in the ischemic area of the myocardium. This finding aligns with the ischemia/reperfusion model, where Metrnl-null mice showed no alterations in inflammatory cell accumulation [40]. However, we found higher levels of circulating CCR2^+^ monocytes in Metrnl-null mice, revealing an inflammatory response after MI that has been previously described in preclinical studies to contribute to maladaptive ventricular remodeling and worsening [26]. Nevertheless, Metrnl cardiac overexpression led to higher levels of circulating CCR5^+^ monocytes with improved cardiac remodeling, consistent with studies showing that CCR5 deficiency is associated with adverse cardiac remodeling after MI [56]. CCR5 signaling has also been shown to play a crucial role in preventing uncontrolled inflammation, recruiting regulatory immune cells, protecting against matrix degradation, and preventing against adverse cardiac remodeling [14].

Moreover, we showed defective vascularization in the border area of infarcted Metrnl-null mice, in agreement with the pro-angiogenic capacity recently described for Metrnl [40, 46, 54]. However, this reduced vascularization was not restored in Metrnl-null mice overexpressing Metrnl, indicating that the protective effects observed in our Metrnl overexpression model are independent of the pro-angiogenic capacity of Metrnl and are more reliant on the modulation of the immune cell response at this time point. Since at day 4 post-MI, vascular remodeling is actively underway [57], the higher CD31⁺ density we observe in the infarct region compared to the border zone likely reflects immature vascular structures, rather than preservation of the original microvasculature.

The immune system plays a multifunctional role throughout the reparative process after injury, regulating both pro- and anti-inflammatory phases. Although Metrnl shows remarkable expression in cardiomyocytes [42], its role as a macrophage-derived cytokine seems more relevant in pathologies associated to regeneration and repair as it has been shown in ischemia/reperfusion models and in skeletal muscle repair [5, 29, 40]. In fact, Metrnl expression has also been linked to inflammatory diseases, such as psoriasis, psoriatic and rheumatoid arthritis, and possibly sepsis [8, 50]. Consistent with this, Metrnl levels correlate to the development and resolution of inflammatory responses in vivo*,* and indeed, Metrnl^−/−^ mice develop inflammatory lesions with age [49].

Moreover, consistent with the previous reports on skeletal muscle, we also observed that macrophages in culture respond to Metrnl [5, 49]. The increased Osm levels observed in response to Metrnl in pro-inflammatory macrophages is very specific and independent of the rest of classical pro-inflammatory markers and mimicked what we observed in vivo. Recently, it has been shown that Osm expression also increases in macrophages in response to exercise-like stimuli [16], indicating that Osm production can be specifically modulated in pro-inflammatory macrophages by external factors, in agreement with our own data.

Osm has a central role during the acute inflammatory phase by regulating the production of secreted proteins by cardiomyocytes such as Reg3β [33]. We demonstrate that, in addition to the chemotactic role of Reg3β on recruiting macrophages to the ischemic area [32,33], Reg3β is responsible to shut down pro-inflammatory macrophage polarization, thus promoting the resolution of the acute inflammatory phase following MI. These data agree with previously published data reporting the anti-inflammatory role of Reg3β during tumor growth [21]. Therefore, we show that the regulation of the Osm–Reg3β axis exerted by Metrnl determines the extent and duration of the acute inflammatory phase post-MI.

The focus on Osm was motivated by our in vitro assays using pro-inflammatory-activated macrophages, in which Osm emerged as the only inflammatory mediator that was consistently and specifically modulated by Metrnl. Importantly, canonical pro-inflammatory mediators typically associated with this macrophage phenotype, including Ccl2, Il6, and Tnfα, remained unchanged following Metrnl treatment. This observation further supports the specificity of the Metrnl-Osm axis rather than a broad suppression of inflammatory signaling. Nevertheless, a limitation of the present study is that we cannot exclude the involvement of additional inflammatory mediators that were not captured in our experimental framework. A more comprehensive and systematic characterization of the inflammatory landscape may therefore represent an important direction for future research. In addition, although our in vivo data are consistent with the proposed mechanism, the regulation of the Osm–Reg3b axis by Metrnl is primarily supported by in vitro evidence, which represents another limitation of the study.

Taken together, we concluded that Metrnl acts as a crucial regulator of the switch from the pro-inflammatory phase to the reparative anti-inflammatory phase. On one hand, Metrnl controls the duration of the pro-inflammatory phase by regulating the Osm–Reg3β axis in cardiomyocyte–macrophage crosstalk. On the other hand, Metrnl directly promotes the anti-inflammatory reparative phase in the myocardium post-MI modulating macrophage trafficking and polarization. Additionally, the identification of a regulatory loop controlling macrophage recruitment and polarization in the ischemic heart offers new opportunities for modulating macrophage phenotypes. This could provide a novel approach to prevent specific deleterious inflammatory effects mediated by macrophage overactivation, while still allowing other phagocytic and reparative responses after MI.

## Supplementary Information

Below is the link to the electronic supplementary material.

Supplementary file1 (TIF 2362 KB)

Supplementary file2 (TIF 6178 KB)

Supplementary file3 (TIF 968 KB)

Supplementary file4 (TIF 3171 KB)

Supplementary file5 (TIF 3465 KB)

Supplementary file6 (TIF 1002 KB)

Supplementary file7 (TIF 288 KB)

Supplementary file8 (docx 46 KB)

## Data Availability

Data will be made available on request. For the sn-RNAseq, data are deposited in Gene Expression Omnibus (GEO) under the accession number GSE335342.
