# Supplementary material for: Meteorin-like regulates cardiac repair via macrophage modulation following myocardial infarction
Source: Basic Res Cardiol. 2026 Jul 31;121(5):987–1010. doi: 10.1007/s00395-026-01203-7 (PMC13593666; doi:10.1007/s00395-026-01203-7)
Supplement: Supplementary file 1 [file 395_2026_1203_MOESM8_ESM.docx]

**Supplemental Information**

**Supplemental Material and Methods**

*Single-cell RNA (scRNA) sequencing data analysis*

Data used for scRNA-sequencing analysis was obtained from the published study from Jung et al. deposited in Gene Expression Omnibus (GEO) under the accession number GSE163129 [1]. Single-cell RNA sequencing data analysis was performed following the code deposited in GitHub by the same researchers (https://github.com/IRCGP-Lab/Macrophage-heterogeneity-after-MI), including some modifications and additional functions to assess a proper filter and characterization of cell populations adapted to our knowledge and aiming. Bioinformatics processing of the scRNA-sequencing data was performed with the R package Seurat (version 3.2.0) [2]. To exclude low quality cells in scRNA-sequencing, we filtered cells with an expressed gene count fewer than 2% or greater than 98%. Additionally, cells in which more than 10% of reads corresponded to mitochondrial genes were removed. Data was log-normalized, and highly variable features were identified based on a variance stabilizing transformation (VST) method. All datasets (steady-state, day 1, 3, 5, and 7) were then integrated using the canonical correlation analysis (CCA) method, “FindIntegrationAnchors” and “IntegrateData” functions in Seurat. Principal components analysis (PCA) was performed on the integrated datasets. Based on the top 40 principal components (PCs), graph-based clustering was performed using the shared nearest neighbor (SNN) modularity optimization with resolution set to 1.4, and all cells were classified into 36 clusters. Clustering data was then applied followed by uniform manifold approximation and projection (UMAP) allowing the visualization of identified clusters in UMAP plots [3]. Each cell cluster was annotated for their cell type using the SingleR R package [4] (version 1.2.4) and/or assessment of well-known cell-type specific markers. Isolated clusters of cells that expressed the endothelial markers (Pecam1 and Kdr) or fibroblast markers (Col1a1 and Col1a2) were removed from further analysis, just as Jung et al. made in their analysis.

Sub-clustering of Macrophages/Monocytes populations was first performed under the same criteria Jung et al. used in their study based on the timepoint where macrophages were majority, resulting in 16 different clusters (Supplemental Figure S6, S7). After that, cells were classified depending on their phenotype (M1, M2, M3, IFN, Proliferating, Ly6c2hi Monocyte, Ly6c2low Monocyte), resulting in the clustering map shown in Figure 1E, F).

To identify significantly differentially expressed genes within each cluster we performed differentially expressed gene (DEG) analysis using the logistic regression test for significance and an average loge fold-change was used as a reference for the identification of macrophage clusters in sub-clustering process and for comparison of average loge fold-change of Ccr2, Osm and Metrnl between all the macrophage/monocyte subclusters. Genes with a positive average loge fold-change value (greater than 0.25) and an adjusted P value lower than 0.05 were kept in the analysis.

**References**

1	Jung SH, Hwang BH, Shin S, *et al.* Spatiotemporal dynamics of macrophage heterogeneity and a potential function of Trem2hi macrophages in infarcted hearts. *Nature Communications 2022 13:1* 2022; 13: 1-15

2	Stuart T, Butler A, Hoffman P, *et al.* Comprehensive Integration of Single-Cell Data. *Cell* 2019; 177: 1888-1902.e21

3	Becht E, McInnes L, Healy J, *et al.* Dimensionality reduction for visualizing single-cell data using UMAP. *Nature Biotechnology 2018 37:1* 2018; 37: 38-44

4	Aran D, Looney AP, Liu L, *et al.* Reference-based analysis of lung single-cell sequencing reveals a transitional profibrotic macrophage. *Nature Immunology 2019 20:2* 2019; 20: 163-172

**Supplemental Figure legends**

**Supplemental Figure S1. Flow cytometry gating strategy**. **(A)** Gating strategy used for phenotyping circulating monocytes. Singlets were gated according to FSC-A/FSC-H, followed by gating monocytes based on the CD45^+^/Ly6G^-^ and F4/80^+^CD11b^+^ populations. Subsequently, proinflammatory monocytes were identified according to Ly6C High population and anti-inflammatory monocytes were identified according to Ly6C Low population. Chemokines receptors (CCR2 and CCR5) were assessed in both populations. The percentages of cells expressing each marker were computed. **(B)** Splenic cells were gated according to their distinctive FSCA/SSC-A appearance from the Singlets population. Macrophages were gated according to CD45^+^/Ly6G^-^ and F4/80^+^CD11b^+^ population. Then, proinflammatory and anti-inflammatory macrophages were gated according to Ly6C expression as previously and CD86. The different % of cells expressing each marker and the median fluorescence intensity (MFI) in each channel were calculated.

**Supplemental Figure S2.** **Single cell RNA-seq (sc-RNAseq) of CD45^+^ myeloid cells from mouse hearts at days 0, 1, 3, 5, and 7 post-MI.** (A) Temporal two-dimensional uniform manifold approximation and projection (UMAP) visualization of cardiac CD45^+^ cells identifying 12 broad cell types. Each point represents a single cell; cell types are color-coded. (B) Temporal feature plot representing the expression levels of *Metrnl.* (C) Temporal two-dimensional uniform manifold approximation and projection (UMAP) visualization of cardiac monocytes/macrophages identifying 7 sub-clusters. Each point represents a single cell; cell types are color-coded. (D) Temporal feature plot representing the expression levels of *Metrnl.*

**Supplemental Figure S3. Survival curve.** 17-weeks old wt, Metrnl^-/-^ mice injected i.p with AAV9-null (Metrnl^-/-^) or with AAV9 carrying Metrnl (AAV9Metrnl) were subjected to permanent coronary artery ligation for 4 days. The percentage survival of mice was represented. No significant differences were observed.

**Supplemental Figure S4**. **Metrnl overexpression enhances cardiac remodelling in wt mice.** (**A**) 17-weeks old wt (white bars) and Metrnl^-/-^ mice injected i.p with AAV9-null (Metrnl^-/-^, grey bars) at baseline were analysed by flow cytometry. Histograms representing the percentage of Ly6C High and Ly6C Low populations in spleen and blood; n=4-5 mice/group. (**B-E**) 17-weeks old wt mice injected i.p with AAV9-null or with AAV9 carrying Metrnl (AAV9Metrnl) were subjected to permanent coronary artery ligation for 4 days. **(B)** *Metrnl* expression levels in the different zones of the myocardium **(C)** Echocardiographic measurements of LVEF, EDV and ESV (**D)** Quantification of remodelling area and immune cell infiltration. Results are expressed as a percentage of total myocardium. **(E)** Gene expression of *Arg1, Osm* and M1/M2 macrophage ratio, expressed as *iNos/Arg1* expression level ratio; n= 5-8 mice/ group. Results are expressed as mean ± SD. Data were analysed by two-way ANOVA (B) or t-test.

**Supplemental Figure S5**. Pro-inflammatory**-macrophage activation induces *Metrnl* and *Osm* expression** (**A**) mRNA levels of genes indicative of anti-inflammatory (Clec10a, Mrc1, Arg1) and pro-inflammatory (Tnf, *Il6,* Ccl2) macrophage activation in RAW264.7 macrophages previously polarized using LPS or IL-4 respectively. (**B**) mRNA levels of *Metrnl* and *Osm* genes in RAW264.7 macrophages previously polarized to pro-inflammatory or anti-inflammatory phenotype. **(C)** mRNA levels of *Kit* in BMDMs. **(D)** *Metrnl* mRNA and protein levels in cell culture media of neonatal cardiomyocytes in culture (NCMs) infected with null adenovirus (CT) or Metrnl adenovirus (AdMetrnl). Data are represented as mean ± SD. ^∗^p < 0.05, ^∗∗^p < 0.01, and ^∗∗∗^p < 0.001 versus controls. Data were analysed by t-test.

**Supplemental Figure S6.** Heatmap showing the top 10 most differentially expressed genes in the 16 sub-clusters identified. Blue and red indicate lower and higher expression, respectively. The typical markers strongly and specifically associated with each sub-cluster are shown on the left.

**Supplemental Figure S7.** Proportion of each sub-cluster among total monocytes/macrophages according to the time-point after MI.
